# Structural studies of crystalline forms of triamterene with carboxylic acid, GRAS and API molecules

**DOI:** 10.1107/S2052252518003317

**Published:** 2018-04-06

**Authors:** Abida Rehman, Amit Delori, David S. Hughes, William Jones

**Affiliations:** a Department of Chemistry, University of Cambridge, Lensfield Road, Cambridge, Cambridgeshire CB2 1EW, England; b Strathclyde Institute of Pharmacy and Biomedical Sciences (SIPBS), University of Strathclyde, 161 Cathedral Street, Glasgow G4 0RE, Scotland

**Keywords:** triamterene, p*K*_a_ analysis, crystal engineering, liquid-assisted grinding, pharmaceutical salt solvates, hydrogen bonding, motif analysis, stable duplex structures

## Abstract

Pharmaceutical salt solvates (dimethyl sulfoxide, DMSO) of the drug triamterene with the coformers acetic, succinic, adipic, pimelic, azelaic and nicotinic acid and ibuprofen are prepared by liquid-assisted grinding and solvent-evaporative crystallization. The modified Δp*K*_a_ rule as proposed by Cruz-Cabeza [(2012 ▸). *CrystEngComm*, **14**, 6362–6365] is in close agreement with the results of this study. All adducts were characterized by X-ray diffraction and thermal analytical techniques, including single-crystal X-ray diffraction, powder X-ray diffraction, differential scanning calorimetry and thermal gravimetric analysis. Hydrogen-bonded motifs combined to form a variety of extended tapes and sheets. Analysis of the crystal structures showed that all adducts existed as salt solvates and contained the amino­pyridinium–carboxyl­ate heterodimer, except for the solvate containing triamterene, ibuprofen and DMSO, as a result of the presence of a strong and stable hemitriamterenium duplex. A search of the Cambridge Structural Database (CSD 5.36, Version 1.18) to determine the frequency of occurrence of the putative supramolecular synthons found in this study showed good agreement with previous work.

## Abstract 2

This work discusses the preparation and characterization of crystalline forms of the drug triamterene with various carboxylic acids including Generally Regarded as Safe and Active Pharmaceutical Ingredients using liquid-assisted grinding and solvent-evaporative crystallization; a method of potential benefit to the pharmaceutical industry. Triamterene is selected as an appropriate model compound because it has poor water solubility which can have an impact on its bioavailability as a drug and it contains numerous hydrogen-bonding sites, thereby allowing a study of the competition between the different potential supramolecular synthons. Cambridge Structural Database studies show good agreement between this and previous studies using similar compounds.

## 1. Introduction   

The compound 2,4,7-tri­amino-6-phenyl­pteridine (C_12_H_11_N_7_), named triamterene (**1**), is a potassium-sparing diuretic and modest inhibitor of di­hydro­folate reductase. It is marketed under the name of Dyrenium (Well Spring Pharmaceutical Corporation, Concordia Pharmaceutical Inc., Carilion Materials Management and GlaxoSmithKline Inc.) for the treatment of oedema (water retention) and, when combined with hydrochlorthiazide as Maxzide (Mylan Pharmaceuticals) and Dyazide (Cardinal Health and SmithKline Beecham), for the treatment of hypertension (high blood pressure).

This drug has poor solubility in water and a low bioavailability (45 µg ml^−1^) (Dittert *et al.*, 1964 ▸), and is therefore an excellent candidate for a cocrystal or salt screen (Thakuria *et al.*, 2013 ▸; Duggirala *et al.*, 2016 ▸). A recent study (Ma *et al.*, 2013 ▸) found two polymorphs of the salt of **1** with cucurbit­[7]uril (CB[7]). The powder of the CB[7] salt forms a stable complex with **1** in aqueous solution with improved dissolution and solubility by a factor of 1.6 > **1** in 0.1 *M* hydro­chloric acid. The increased solubility and consequent oral bioavailability of **1** in this case was attributed to the formation of a hydro­philic cylinder composed of two hydro­philic portals containing carbonyl functional groups, in which two molecules of **1** are encapsulated to form a stable complex. As an alternative approach, adduct (cocrystal or salt) formation with carb­oxy­lic acids (Bhatt *et al.*, 2009 ▸), Generally Regarded As Safe (GRAS) molecules (Delori *et al.*, 2008 ▸; Aitipamula *et al.*, 2014 ▸; Lu & Rohani, 2010 ▸) and Active Pharmaceutical Ingredients (APIs) (Huang *et al.*, 2014 ▸; Sowa *et al.*, 2012 ▸; Báthori *et al.*, 2011 ▸; Delori, Galek *et al.*, 2013 ▸; Grobelny *et al.*, 2011 ▸) also provides a means to improve the physicochemical properties of **1**. For example, a recent study by Li *et al.* (2015 ▸) reported enhanced solubility and dissolution as a result of salt formation with dl-mandelic acid and saccharin.

Adduct (cocrystal or salt) formation reactions do not only find application in modifying solubility and dissolution profiles (Aitipamula, Vangala *et al.*, 2012 ▸; Aitipamula, Wong *et al.*, 2012 ▸; Sanphui *et al.*, 2011 ▸) but have also been used in altering various other physical properties, such as colour (Stahl & Wermuth, 2008 ▸; Pallipurath *et al.*, 2015 ▸; Delori *et al.*, 2016 ▸), melting point (Friščić & Jones, 2010 ▸), stability to humidity (Trask *et al.*, 2005 ▸, 2006 ▸), tableting properties (Bučar *et al.*, 2015 ▸) fluorescence (Bučar *et al.*, 2013 ▸; Yan *et al.*, 2013 ▸) and polymorphic conversion (Delori *et al.*, 2014 ▸).

**Figure 1:**
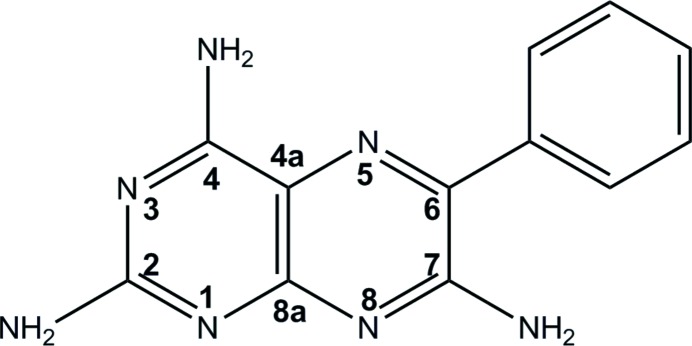
Chemical diagram of **1** labelled with the numbering scheme that is used throughout this manuscript.

As shown in Fig. 1 ▸, the 2-, 4- and 7-amino groups of the pteridine ring of **1** have good hydrogen-bond donor ability, while the N atoms at positions 1, 3, 5 and 8 can act as potential hydrogen-bond acceptors; however, the N atoms of the amino groups can also act as acceptors, making a total of six donors and seven potential hydrogen-bond acceptors. Since **1** has a large number of potential hydrogen-bond acceptor and donor sites, as well as the option of ionization to form a salt, it is particularly interesting from a crystal engineering standpoint. Furthermore, the cocrystal/salt chemistry of **1** is not well understood, making it a suitable candidate for further experimental work.

Within the literature, different strategies have been adopted for the selection of suitable pharmaceutical coformers. One method is based on the hydrogen-bond propensity tool (Bhatt *et al.*, 2009 ▸; Delori *et al.*, 2012 ▸; Delori, Galek *et al.*, 2013 ▸; Huang *et al.*, 2014 ▸; Wood *et al.*, 2014 ▸) developed by the Cambridge Crystallographic Data Centre (CCDC) to predict the possibility of new polymorphs or adducts based on observed hydrogen bonding in chemically related molecules. Putative pharmaceutical adducts have also been predicted using the CCDC software *GOLD* (Galek *et al.*, 2007 ▸, 2010 ▸). A further approach based on molecular shape and polarity descriptors, as a statistical analysis of the Cambridge Structural Database (CSD), found these properties to be the most influential in successful crystal formation (Fábián, 2009 ▸). In the present study, we select two classes of coformer for initial screening: mono- and di­carb­oxy­lic acids. Within each of these categories, diversity is created using molecular weight, alkyl or phenyl character and varying the number of available hydrogen-bond donors or acceptors (see Fig. *S*1 in the supporting information).

**Figure 2:**
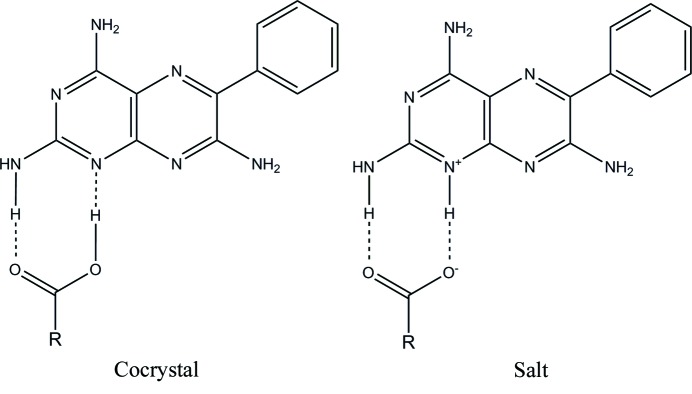
Proposed supramolecular heterosynthons between **1** and the acid coformers.

The supramolecular synthon approach is widely used in the search for potential cocrystal or salt formation (Desiraju, 1995 ▸; Varughese *et al.*, 2010 ▸; Arhangelskis *et al.*, 2012 ▸). This approach relies on the interaction between complementary functional groups in the formation of hydrogen-bonded adducts (Etter, 1991 ▸; Etter & Reutzel, 1991 ▸; Etter *et al.*, 1990 ▸). In this study, the N1 atom and the 2-amino group in a molecule of **1** are predicted to interact with the carboxyl functional group of the carb­oxy­lic acid. The predicted 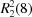 synthon will either involve N—H⋯O and O—H⋯N (both neutral) hydrogen bonds to form a cocrystal or N^+^—H⋯O^−^ (charge assisted) and N—H⋯O (neutral) hydrogen bonds to form a salt (see Fig. 2 ▸).

The exact nature of the complex formed is difficult to predict using only synthon information, details of the molecular recognition process (including kinetics) must also be considered. For this reason, the Δp*K*_a_ rule was applied by Cheney, Shan *et al.* (2010 ▸) and Cheney, Weyna *et al.* (2010 ▸). It is widely acknowledged (Childs *et al.*, 2007 ▸) that if the calculated Δp*K*_a_ lies in the range 0–3 then prediction becomes unreliable. An improvement of this method has subsequently been proposed by Cruz-Cabeza (2012 ▸) and this method is explored in greater detail during this study.

## 2. Experimental   

Pharmaceutical salt solvates (DMSO) of the drug triamterene with the coformers (**a**) acetic, (**b**) succinic, (**c**) adipic, (**d**) pimelic, (**e**) azelaic and (**f**) nicotinic acid, and (**g**) ibuprofen are prepared by liquid-assisted grinding (LAG) and solvent-evaporative crystallization (SEC). All chemicals were purchased from Sigma–Aldrich and used without further purification. The solvents employed for the crystallizations were spectroscopy grade of the highest purity available.

### 2.1. Adduct prediction   

The method proposed by Cruz-Cabeza (2012 ▸) is explored in order to predict the outcome of a given adduct formation reaction where *P*_obs_ is the probability (%) of observing *AB* (cocrystal) and *A* ^−^ *B* ^+^ (salt).
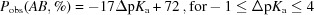


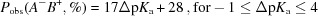
The *P*_obs_ values for the proposed adduct formation reactions were calculated using the above formulae and compared with the results obtained by experiment in Table *S*1.

### 2.2. Liquid-assisted grinding   

LAG experiments were performed by placing 200 mg of a physical mixture of **1** with the corresponding acid (**a**–**e**) in a 1:1 or 2:1 stoichiometric ratio in a 15 ml stainless-steel grinding jar, accompanied by the addition of 30–50 µl of solvent (DMSO). Grinding was carried out using a Retsch MM200 mixer mill with a frequency of 30 Hz for 30 min together with two stainless steel grinding balls of 7 mm diameter.

### 2.3. Solvent-evaporative crystallization   

Crystals of pure triamterene were obtained by dissolving 10 mg of **1** in 30 ml of methanol and the solution was then heated and filtered prior to SEC. The solutions for the crystallization of **1a**–**g**·DMSO were prepared by dissolving the product of the LAG process (∼50 mg) in DMSO (2 ml) and to aid dissolution, the contents were sonicated prior to gentle heating in a bead bath at 323 K and were filtered (if necessary) into a suitable vial. Colourless high-quality crystals suitable for single-crystal X-ray diffraction analysis (SCXRD) were obtained in all cases by slow evaporation of the methanol or DMSO solution at room temperature for 3–7 d (see Table *S*2 for a summary of the crystallization data).

### 2.4. Single-crystal X-ray diffraction   

A search of the CSD (5.36, Version 1.18; Groom *et al.*, 2016 ▸) revealed two crystal structures of **1** with the refcodes FITZAJ (Schwalbe & Williams, 1987 ▸) and FITZAJ01 (Tutughamiarso & Bolte, 2007 ▸). These crystal structures have fairly high *R*_1_ values [*F*_o_ > 3σ and *I* > 2σ(*I*) of 0.09 and 0.0739, respectively] so in our previous work (Hughes *et al.*, 2017 ▸), we redetermined the crystal structure of **1** (CCDC deposition code: 1532364) using a crystal grown from methanol. CCDC deposit 1532364 forms the basis for the description of the crystal structure of **1** that follows.

Experimentally, good-quality single crystals of **1a**–**g**·DMSO were chosen by selection under an optical microscope, glued to a glass fibre and mounted on the goniometer of a Bruker diffractometer equipped with an APEX CCD detector at low temperature (150 K for **1** **d**·DMSO, 180 K for **1** **a**·DMSO, **1** **c**·DMSO, **1** **e**·DMSO, **1** **f**·DMSO and **1** **g**·DMSO and 220 K for **1** **b**·DMSO) and graphite-monochromated Mo *Kα* radiation (*λ* = 0.71073 Å). In all cases, the data were collected without complication and all crystals were found to be stable throughout the collection period. Intensity data were processed using *SAINT* (Bruker, 2007 ▸), followed by an absorption correction using *SADABS* (Bruker, 2001 ▸). The structures for **1** **a**–**g**·DMSO were all solved using *SIR92* (Altomare *et al.*, 1993 ▸) and were refined using the least-squares methods employed in *SHELXL2013* (Sheldrick, 2015 ▸). All non-H atoms were refined using anisotropic methods, the H atoms either placed in calculated positions by refinement or found by experiment (electron density) where appropriate. The position of the H atom on ring atom N1 was of particular importance and found by experiment in all cases except **1** **d**·DMSO, as the experimental data for this crystal was poor. All structure refinements converged to good *R* factors and hydrogen bonds were determined using *PLATON* software (Spek, 2009 ▸). All diagrams relating to the crystal structure of **1** **a**–**g**·DMSO were generated using the *Mercury 3.9* module of the CSD Enterprise package (Macrae *et al.*, 2008 ▸).

**Figure 3:**
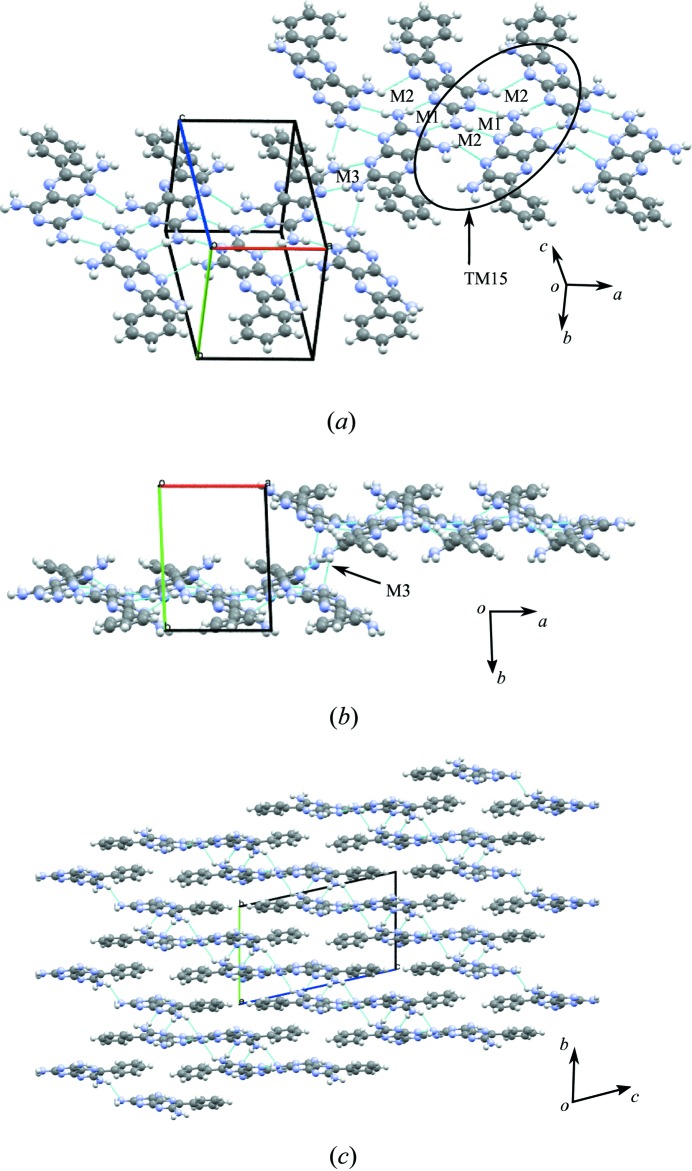
(*a*) Motifs M2, M1 and M2 (tape motif TM15) and motif M3 observed in **1**. (*b*) The view along *c* illustrating the relationship between adjacent tapes using the M3 motif (side view). (*c*) Packing diagram of the stepped hydrogen-bonded sheets.

**Figure 4:**
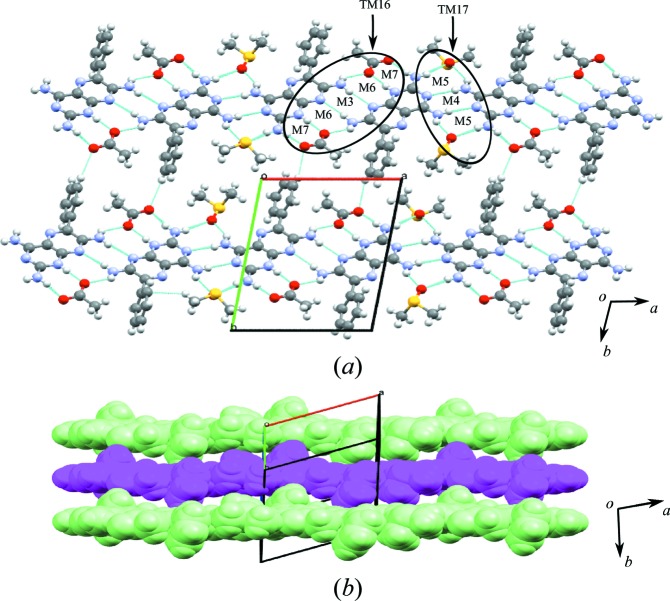
(*a*) Motifs M7, M6, M3, M6 and M7 (TM16), and M5, M4 and M5 (TM17) found in the crystal structure of **1a**·DMSO. (*b*) The host–guest network in the form of stepped tapes forming a sheet structure.

**Figure 5:**
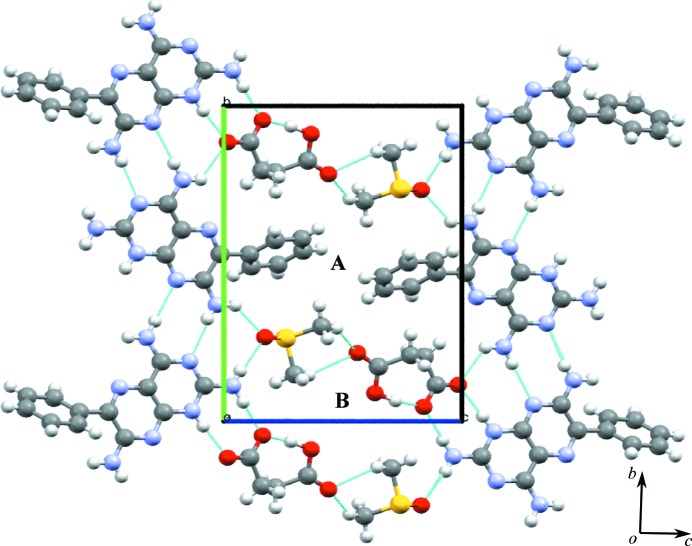
The cyclic hydrogen-bonded networks **A** and **B** of the crystal structure of **1b**·DMSO.

**Figure 6:**
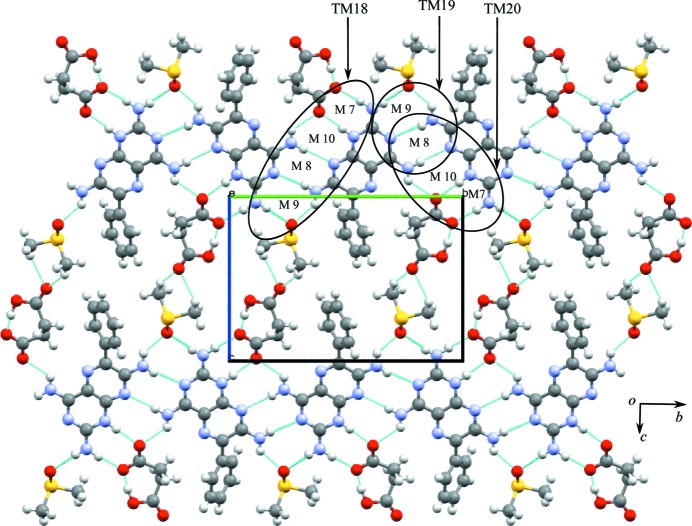
Motifs M7, M10, M8 and M9 (TM18), M8 and M9 (TM19), and M7, M10 and M8 (TM20) observed in the hydrogen-bonded sheets of **1b**·DMSO.

**Figure 7:**
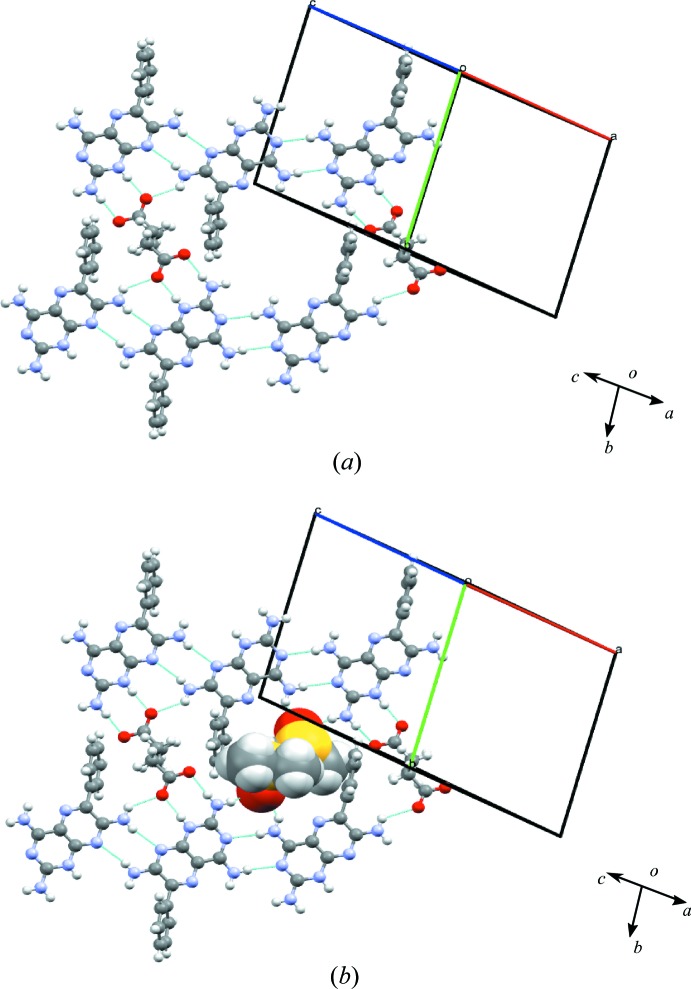
(*a*) The eight-membered hydrogen-bonded cyclic host network with a cavity of 15.3 × 10.0 Å dimensions and (*b*) the cavity filled with two DMSO molecules as guests in **1c**·DMSO.

**Figure 8:**
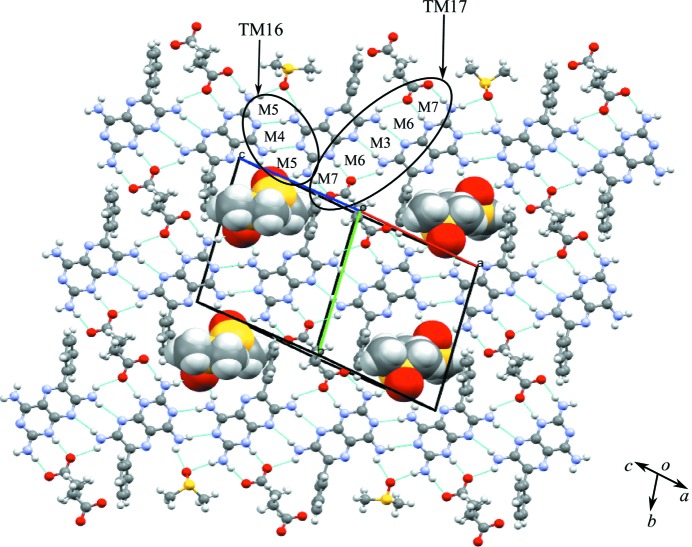
Motifs M5, M4 and M5 (TM16), and M7, M6, M3, M6 and M7 (TM17) found in the hydrogen-bonded sheets of **1c**·DMSO.

**Figure 9:**
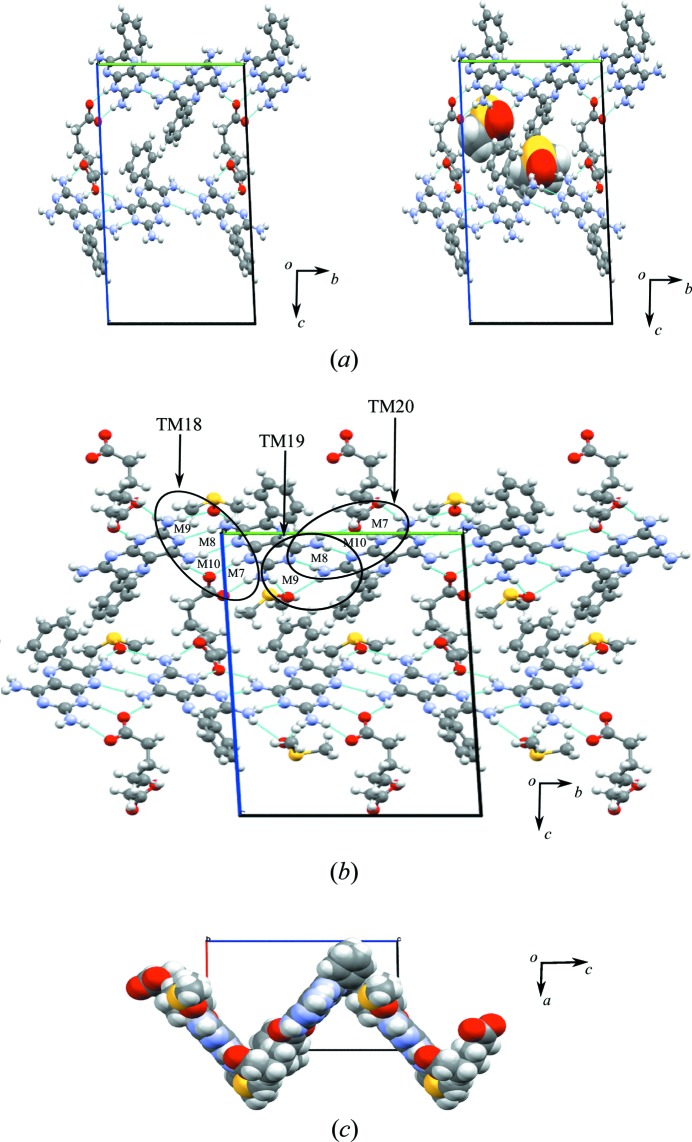
(*a*) The eight-membered hydrogen-bonded host network with a cavity of 14.9 × 9.1 Å and the same cavity filled with two DMSO molecules as guests. (*b*) Motifs M7, M10, M8 and M9 (TM18), M8 and M9 (TM19), and M7, M10 and M8 (TM20) found in the sheet structure formed by the host–guest hydrogen-bonded network. (*c*) The zigzag nature of the sheet structure seen in **1d**·DMSO.

**Figure 10:**
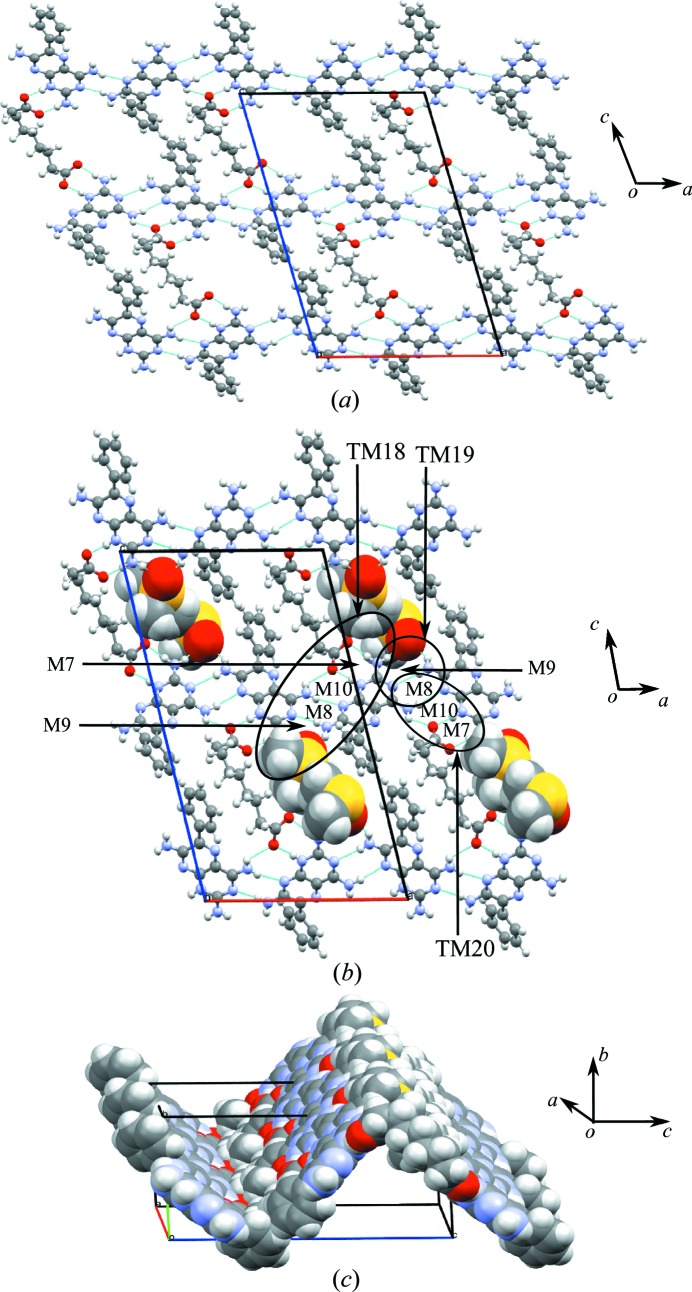
(*a*) The cyclic hydrogen-bonded host network. (*b*) The same network with each cavity filled with two DMSO molecules showing the motifs M7, M10, M8 and M9 (TM18), M8 and M9 (TM19), and M7, M10 and M8 (TM20). (*c*) The space-filling model showing the zigzag nature of the **1e**·DMSO sheets.

**Figure 11:**
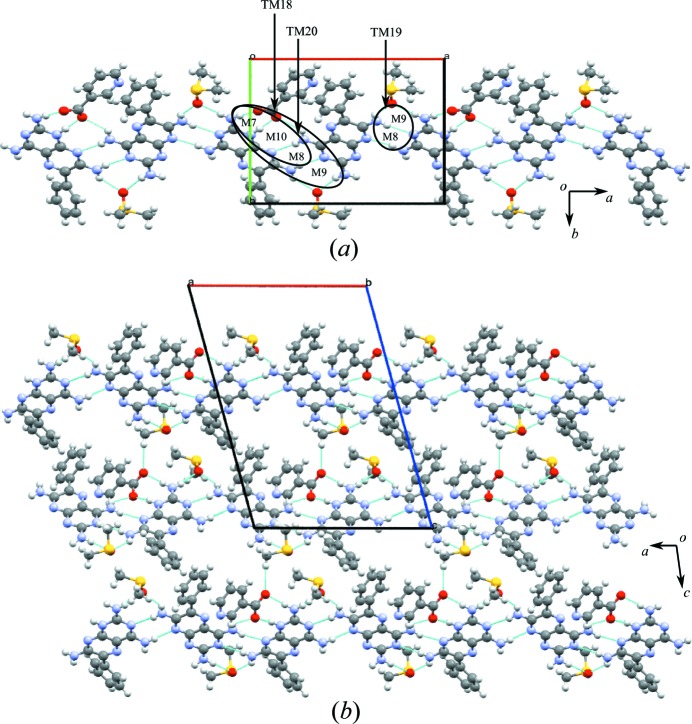
(*a*) The motifs M7, M10, M8 and M9 (TM18), M8 and M9 (TM19), and M7, M10 and M8 (TM20) that create the hydrogen-bonded tape and (*b*) sheet that forms part of the crystal structure of **1f**·DMSO.

**Figure 12:**
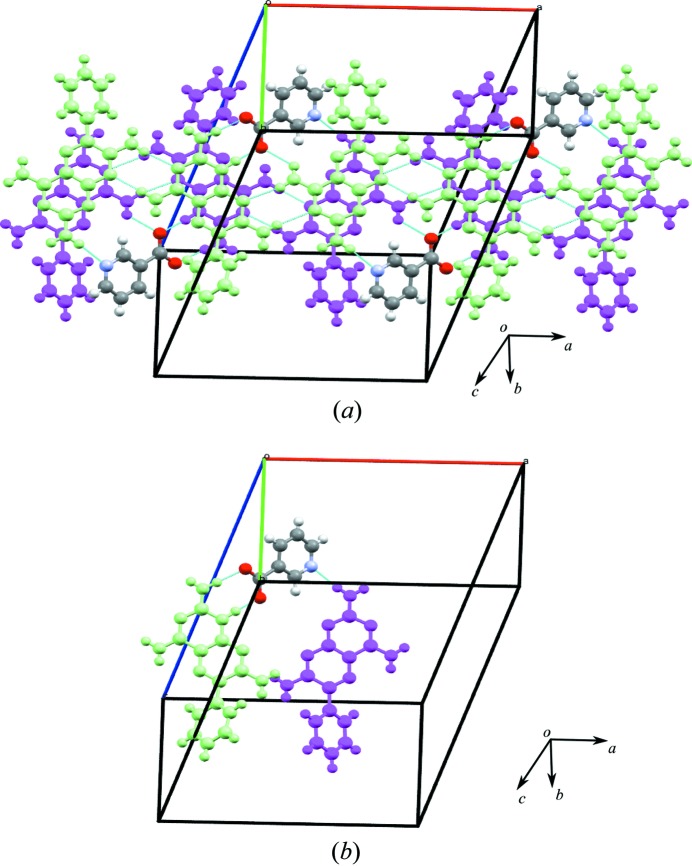
(*a*) Stacking of molecules of **1** to form double hydrogen-bonded chains through (*b*) nicotinic acid linkages in **1f**·DMSO.

**Figure 13:**
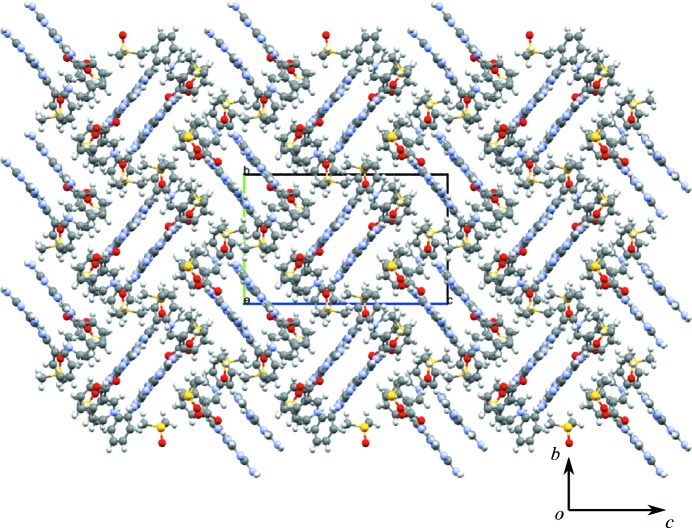
The three-dimensional packing diagram to show the molecules of **1** arranged as zigzag double ribbons in the crystal structure of **1f**·DMSO.

**Figure 14:**
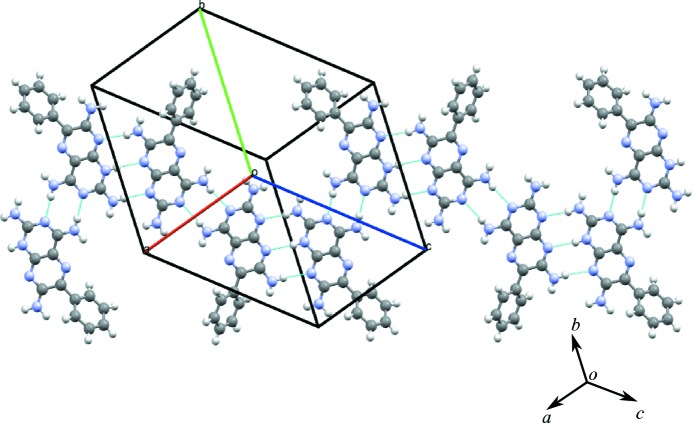
The crinkled tape of molecules of **1** observed in the crystal structure of **1g**·DMSO.

**Figure 15:**
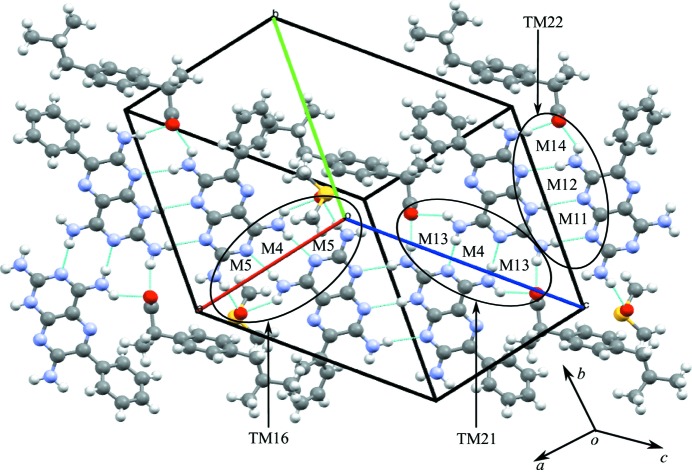
The crinkled tape of molecules of **1** with molecules of coformer **g** and solvent DMSO with the motifs M5, M4 and M5 (TM16), M13, M4 and M13 (TM21), M11, M12 and M14 (TM22), and M11 and M12 (TM23) in the crystal structure of **1g**·DMSO.

**Figure 16:**
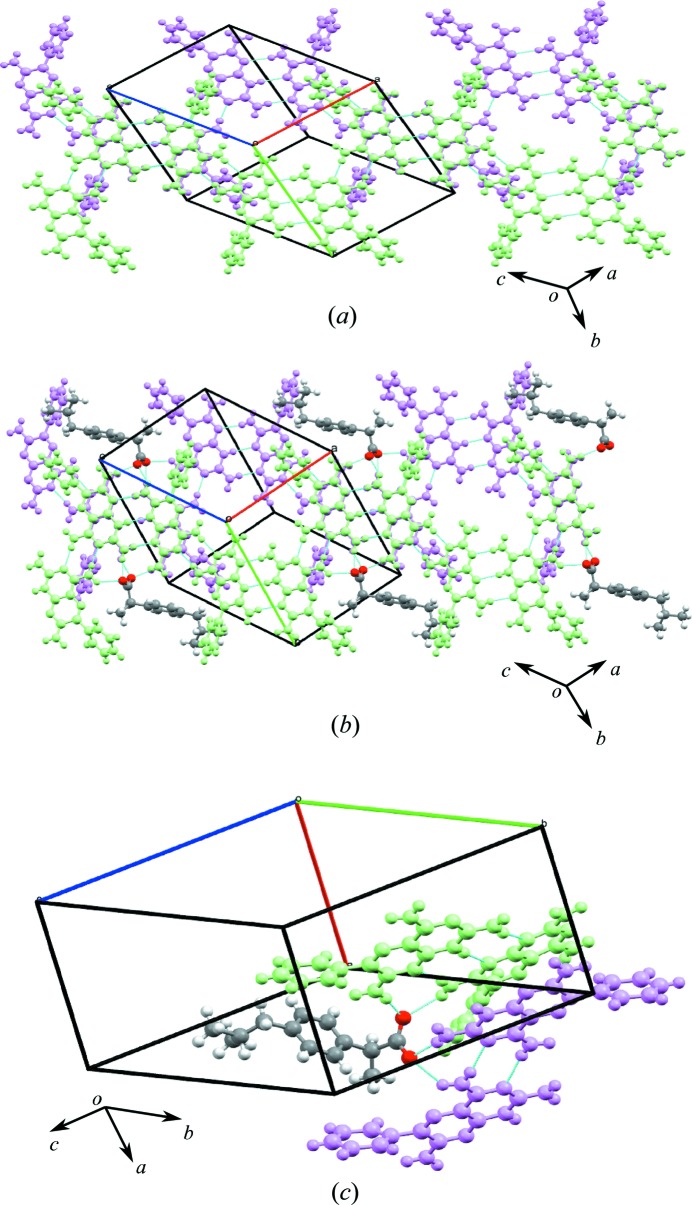
(*a*) Stacking of molecules of **1**, (*b*) the linking of molecules of **1** using molecules of **g** and (*c*) the specific interactions observed between the stacked hydrogen-bonded layers of **1** and **g** as seen in the crystal structure of **1g**·DMSO.

Table 1 ▸ contains selected crystallographic parameters for **1** and **1** **a**–**g**·DMSO. Table *S*3 (see supporting information) contains further crystallographic details relating to data collection and structure refinement for **1** and **1** **a**–**g**·DMSO. Table *S*4 contains details of the labelling scheme for **1** and **1** **a**–**g**·DMSO, Figs. *S*2–*S*9 show the asymmetric units for **1** and **1** **a**–**g**·DMSO and Tables *S*5–*S*12 contain details of the hydrogen bond schemes for **1** and **1** **a**–**g**·DMSO. The supplementary crystallographic data (CIF files) for **1a**–**g**·DMSO are available in the supporting information for this article or from the Cambridge Crystallographic Data Centre. The supporting information also contains enhanced three-dimensional interactive structural files in *Mercury* (MRYX) format corresponding to Figs. 3–16 in the manuscript.

### 2.5. Powder X-ray diffraction   

Powder X-ray diffraction (PXRD) data were collected on a Phillips PW3710 diffractometer with nickel-filtered Cu *K*α radiation (1.5406 Å) at 40 kV and 40 mA using a scanning RTMS X’Celerator detector. The samples were prepared by gently pressing on a glass slide with a sample groove. Data were collected between 3 ≤ 2θ ≤ 50° at ambient temperature with a step size of 0.0167° at a time per step of 13.97 s for a total scan time of 5 min.

To show that the crystals selected for single-crystal analysis were representative of the bulk powders obtained by LAG, the experimental patterns were compared with the simulated patterns for samples **1** **a**–**g**·DMSO (see Figs. *S*10–*S*16 of the supporting information for reference).

### 2.6. Differential scanning calorimetry measurements   

For differential scanning calorimetry (DSC) analysis, a standard 40 µl aluminium pan was used by placing 3–7 mg of gently ground material such that the bottom of the pan was uniformly covered with sample. The pan was sealed with an aluminium lid using a press and a small hole made in the lid. A Mettler Toledo DSC 822 instrument with an 80 cm^3^ min^−1^ nitro­gen purge flow rate was used for the analysis. The temperature range used was 298–673 K with a scan rate of 10 K min^−1^. Mettler Toledo *STARe* software (Version 8) was used for the analysis of thermal data obtained.

DSC results for sample **1** are shown for reference in Fig. *S*17 (see supporting information).

### 2.7. Thermometric gravimetric analysis measurements   

Samples were prepared by placing 7–13 mg of material in a standard 100 µl aluminium pan. A Mettler Toledo TGA/ SDTA851e/SF/1100 instrument with an air purge flow rate of 50 cm^3^ min^−1^ was used for the thermogravimetric analysis (TGA). The samples were heated at a rate of 10 K min^−1^ between 303–673 K and a data analysis was carried out using Mettler Toledo *STARe* software (Version 8).

DSC and TGA curves for samples **1** **a**–**g**·DMSO are shown in Figs. *S*18–*S*24 (supporting information).

## 3. Results and discussion   

All the crystalline adducts in the present study between **1** and coformers **a**–**g** existed as salt solvates (Grothe *et al.*, 2016 ▸). The salt/coformer predictions are summarized in Table *S*1 and the results for all the predicted crystalline adducts were in good agreement with the experiment except for **1a**·DMSO and **1g**·DMSO where the Δp*K*_a_ values (Hilal *et al.*, 1995 ▸) are smallest, thereby providing support for the validity of the modified p*K*_a_ rule of Cruz-Cabeza (2012 ▸) in this case. Exceptions are known to the p*K*_a_ rule, primarily because of the fact that p*K*_a_ is a measure of acidity in aqueous solution and the acidity of the same molecules in the crystalline state may be different (Molčanov & Kojić-Prodić, 2010 ▸).

A solvent screen revealed **1** to be generally insoluble in most common solvents and it also has a low solubility in most solvents compared with the coformers **a**–**g** chosen for this study. LAG experiments using methanol failed since single crystals could not be obtained and the melt cocrystallization experiments were unsuccessful because of the high melting point difference between **1** and the coformers **a**–**g**. For these reasons, DMSO was employed as the solvent of choice for the LAG and SEC experiments described in this study since it could be used in relatively small amounts (LAG, 30–50 µl and SEC, 2 ml) and yielded crystalline batches that showed good reproducibility on this scale (see Table *S*2 for further details).

To enable accurate comparison of the crystal structures, a consistent numbering scheme is used in this study and is shown in Fig. 1 ▸. If proton transfer occurs then, from the p*K*_a_ values, the transfer is expected to occur from the carb­oxy­lic acid group H1X to the most basic ring nitro­gen, N1, of **1**. The numbering scheme for molecules of **1** follow IUPAC recommendations for pteridine-like molecules, whilst individual molecules of **1**, coformer and solvent are labelled according to IUCr rules (see Table *S*4 for details). All asymmetric units and lists of hydrogen bonds are included in Figs. *S*2–*S*9 and Tables *S*5–*S*12, respectively.

All comparisons of experimental PXRD and simulated PXRD from SCXRD agree closely (see Figs. *S*10–*S*12 and *S*14–*S*16) except for **1d**·DMSO (see Fig. *S*13) and this is thought to be a result of the preferred orientation of the plate-like crystals noted in this sample.

In an earlier paper (Hughes *et al.*, 2017 ▸), we performed a detailed analysis of the crystal structure of **1**. We have used this knowledge to create a refined version of the graph-set analysis that can describe the similarities and differences between the crystal structures obtained in this study. Crucial to this refinement of graph-set analysis is the concept of the motif which may be defined as a characteristic pattern of hydrogen bonds within the crystal structure. These interactions may be intermolecular, intramolecular or a combination of the two. Motifs are sets of interactions between functional groups creating a particular pattern, *e.g.* rings, chains or discrete contacts. Examples of hydrogen-bonding motifs include the common carb­oxy­lic acid ring dimer, the N—H⋯N chain, and the amide ring dimer. As an extension of this concept, we have introduced the term ‘tape motif’ to include groups of two or more individual motifs and supramolecular synthons defined by Desiraju (1995 ▸) as ‘structural units within supermolecules which can be formed and/or assembled by known or conceivable synthetic operations involving intermolecular interactions’. Using these concepts we are able to describe the crystal structure with the view of recognizing the important similarities and differences that exist within the group of structures chosen for this study and beyond.

### 3.1. The crystal structure of triamterene   

The single-crystal data obtained from our earlier paper demonstrates that **1** crystallizes in the triclinic space group 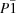 with two independent molecules in the asymmetric unit (*Z* = 4), as shown in Table 1 ▸. The asymmetric unit of **1** is shown in Fig. *S*2 and a list of hydrogen bonds is given in Table *S*5.

The crystal structure shows molecules of *A* and *B* linked into tapes by N—H⋯N hydrogen bonds between the H atoms of the 2-amino groups and the N1 and N3 atoms of adjacent molecules, resulting in the formation of the M1 motif with graph-set notation 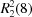. Additionally, the M2 motif with graph-set notation 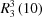 exists from the formation of three N—H⋯N hydrogen bonds using the N atoms at positions 1 and 8 interacting with the 2- and 4-amino groups of three molecules of **1** (see Fig. 3 ▸ *a*). The M3 motif joins two molecules in adjacent tapes through N—H⋯N hydrogen bonds involving the 7-amino group (see Fig. 3 ▸ *b*). It is worth noting that although M1 and M3 motifs are both 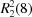 homosynthons, they are numbered differently as they occupy different positions on the component molecules of **1**.

Other ring motifs are present (which are not labelled) that satisfy the hydrogen-bond donor and acceptor requirements of **1**, but for the purposes of simplification, only the motifs that occur repeatedly in the subsequent adducts of **1** are considered in this discussion. The motifs M2, M1 and M2 combine to form the tape motif TM15 and the hydrogen-bonded tapes seen in the crystal structure of **1** (see Fig. 3 ▸ *a* for details).

Overlapping tapes of **1** (Fig. 3 ▸ *b*) create a stepped hydrogen-bonded sheet using the M3 motif and these adjacent hydrogen-bonded sheets pack to form the full three-dimensional crystal structure (as seen in Fig. 3 ▸ *c*).

### 3.2. Salt solvate of triamterene with acetic acid and DMSO (**1a**·DMSO)   

Crystals of adduct **1a**·DMSO are obtained from a mixture of **1** and **a** (1:1) in a solution of DMSO to create a crystalline salt solvate in the triclinic space group 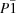 (see Table 1 ▸). The asymmetric unit contains two molecules each of **1**, coformer (carb­oxy­lic acid) and DMSO. The asymmetric unit of **1a**·DMSO is shown in Fig. *S*3 and a table of hydrogen bonds is included (see Table *S*6).

The crystal structure shows molecules of **1** interacting with each other *via* N—H⋯N hydrogen bonds, creating the M3 and M4 motifs, both with the graph-set notation 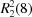. **1** is also protonated at the most basic atom (N1) and the complementary groups on **1** and **a** interact through charge-assisted N^+^—H⋯O^−^ and neutral N—H⋯O hydrogen bonds, creating the M7 motif, also with graph-set notation 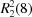. DMSO interacts with two molecules of **1** *via* two N—H⋯O hydrogen bonds, in addition to an intermolecular N—H⋯N bond between adjacent molecules of **1**, which creates the M5 motif with 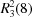 graph-set notation. Motifs M7, M6, M3, M6 and M7 combine to form TM16, and M5, M4 and M5 combine to form TM17 (see Fig. 4 ▸ *a* for details).

These tape motifs create a supramolecular tape of **1** molecules (containing additional acid and solvent molecules) that is interlinked to an adjacent tape of **1** through (C—H⋯O) weak hydrogen bonds that create voids in the crystal structure (Fig. 4 ▸ *a*). Around each void, hydrogen-bonded molecules of **1** create **A** and **B** cyclic networks. In the **A** cyclic network, two DMSO molecules occupy the void; however, in the **B** network, the acid molecules protrude into the void.

The expansion of these combined (**A** and **B**) cyclic networks in two dimensions results in the formation of a hydrogen-bonded sheet formed by stepped tapes similar to that seen in **1** and shown in Fig. 4 ▸(*b*) using a space-filling representation.

### 3.3. Salt solvate of triamterene with succinic acid and DMSO (**1b**·DMSO)   

**1b**·DMSO crystallizes from DMSO solution as a salt solvate in the monoclinic *P*2_1_/*c* space group (see Table 1 ▸). The asymmetric unit contains one molecule each of **1, b** and DMSO (1:1:1). The asymmetric unit of **1b**·DMSO is shown in Fig. *S*4 and the hydrogen bonds are given in Table *S*7. It can be seen from the asymmetric unit that only one of the two carb­oxy­lic acid groups of **b** is deprotonated and the H atom is transferred to the most basic atom (N1) of **1**; the other carboxyl group is involved in an intramolecular O—H⋯O^−^ hydrogen bond and is therefore not available for deproton­ation.

The crystal structure of **1b**·DMSO arises from the formation of two types of hydrogen-bonded network, namely **A**, which is a ten-membered cyclic hydrogen-bonded network, and **B**, a six-membered cyclic hydrogen-bonded network similar to that seen in **1a**·DMSO. Furthermore, the **A** network involves two sets of three molecules (trimers) of **1** linked by the M8 motif that interact with two **b** molecules through the M7 motif present in **1a**·DMSO (see Fig. 5 ▸). Two DMSO molecules act as a bridge between **1** and **b** creating the M9 motif and completing the ten-membered cyclic network. The cavity created by the **A** network is filled by two phenyl rings of **1** and, if these phenyl rings are flipped away from the cavity, voids of 12.7 × 9.0 Å are created. In cyclic network **B**, two single molecules of **1** connect with two molecules of **b**, creating two M7 motifs, and the two DMSO molecules protrude into the cavity formed by **1**, with **b** acting as a bridge, joined to **1** by hydrogen bonds and completing the six-membered cyclic network (Fig. 5 ▸). We note that in the crystal structure the cyclic networks are made by **1**, **b** and solvent molecules (Fig. 5 ▸), as opposed to only **1** and **a** in **1a**·DMSO. Motifs M7, M10, M8 and M9 combine to create the tape motif TM18, M8 and M9 form TM19, and M7, M10 and M8 form TM20, and so complete the hydrogen-bond description of the tape structure found in **1b**·DMSO.

Further expansion of the hydrogen-bonded tape structure in the second dimension results in a sheet structure in which rows of M8 motifs of **1** are interconnected through carb­oxy­lic acid and solvent molecules by weak hydrogen bonds (see Fig. 6 ▸ for details).

### 3.4. Salt solvate of triamterene with adipic acid and DMSO (1c·DMSO)   

Crystallization of **1** and **c** from DMSO generated a 2:1 salt (2**1** ^+^·**c** ^2−^) together with two molecules of DMSO in the triclinic space group 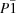 (see Table 1 ▸); **c** acts as a proton donor by the deprotonation of both protons of the carb­oxy­lic acids. The asymmetric unit is shown in Fig. *S*5 and contains two molecules of **1**, one of **c** and two of DMSO (2:1:2). One DMSO molecule is found to be disordered over two sites with occupancies of 0.7 and 0.3. For the purposes of the crystal structure refinement, geometric constraints and a common isotropic displacement parameter for the non-H atoms were applied to both. A list of hydrogen bonds for **1c**·DMSO is given in Table *S*8.

The crystal structure is a host–guest assembly, with the framework created by molecules of **1** and **c**, as shown in Figs. 7 ▸(*a*) and 7(*b*). The interaction between **1** and **c** is through the M7 motif seen in earlier structures, while the molecules of **1** interlink through M3 and M4 motifs (also present in **1a**·DMSO) to form two sets of three molecules (trimers) which link to form sheets. The trimers (tapes) also connect with each other through two acid molecules by hydrogen bonding. This results in the formation of an eight-membered cyclic host network with a cavity of 10.0 × 15.3 Å containing the two molecules of DMSO, as shown in Fig. 7 ▸(*b*).

Motifs M5, M4 and M5 (TM16), and M7, M6, M3, M6 and M7 (TM17) create the hydrogen-bonded network structure (see Fig. 8 ▸). Such host–guest assemblies are well known in the field of supramolecular chemistry (Bhatt *et al.*, 2009 ▸; Galcera *et al.*, 2012 ▸).

### 3.5. Salt solvate of triamterene with pimelic acid (**1d**·DMSO)   

The molecular adduct of **1d**·DMSO crystallizes in the triclinic 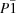 space group (see Table 1 ▸) as a salt between **1** and **d** in which the acid is found to be doubly deprotonated, transferring protons to two separate molecules of the most basic atom (N1) of **1**. The asymmetric unit of the crystal structure is shown in Fig. *S*6 and contains four molecules of **1** (*A*, *B*, *C* and *D*), two molecules of **d** (*E* and *F*) and two molecules of DMSO (*G*). Hydrogen-bond details for **1d**·DMSO are given in Table *S*9.

In the crystal structure, molecules of **1** are connected *via* N—H⋯N hydrogen bonds joined by M8 motifs to form trimers. The acid molecules act as spacers between the trimers joined by the M7 motif and create a zigzag crinkled supramolecular sheet (Fig. 9 ▸ *a*), as seen in **1b**·DMSO (see Fig. 6 ▸). As noted earlier, this tape is fundamentally different from the supramolecular tape found in **1c**·DMSO (see Fig. 7 ▸) since, in this case, motifs M3 and M4 are not involved in creating a trimer of **1** molecules.

Expansion of the supramolecular tape in the second dimension results in a cyclic eight-membered host network, creating a cavity of 14.9 × 9.1 Å (Fig. 9 ▸ *a*). This cavity is filled with two DMSO molecules that act as guests joined to the tapes by M9 motifs with the phenyl rings of **1** also extending into the space (Fig. 9 ▸ *a*). Motifs M7, M10, M8 and M9 (TM18), M8 and M9 (TM19), and M7, M10 and M8 (TM20) are also found in the hydrogen-bonded sheet (see Fig. 9 ▸ *b*).

A hydrogen-bonded sheet structure formed by the combination of host–guest networks is shown in Fig. 9 ▸(*c*) in which molecular tapes of M8 motifs interconnect with M7 motifs with the acid. Tape motifs TM18, TM19 and TM20 are also found in the hydrogen-bonded sheet seen earlier in **1b**·DMSO.

The zigzag stacking of the molecules in this structure is represented by a space-filling model (viewed along the *b* axis) and is shown in Fig. 9 ▸(*c*).

### 3.6. Salt solvate of triamterene with azelaic acid (**1e**·DMSO)   

The salt solvate **1e**·DMSO is obtained from a DMSO solution by dissolving the LAG product of **1** and **e** in a 2:1 ratio. The asymmetric unit of the resulting crystal structure is shown in Fig. *S*7 and contains two molecules each of **1** and DMSO along with one **e** acid molecule in the monoclinic space group *P*2_1_/*n* (see Table 1 ▸). Both –COOH groups of azelaic acid are deprotonated, as seen in **1c**·DMSO and **1d**·DMSO. Hydrogen bond details for **1e**·DMSO are given in Table *S*10.

The crystal structure shows molecules of **1e**·DMSO form a hydrogen-bonded sheet. Further analysis of the sheet shows that molecules of **1** are joined by M8 motifs to form tapes, as shown in Fig. 10 ▸(*a*). These tapes are held together by molecules of azelaic acid through M7 motifs utilizing charge-assisted N^+^—H⋯O^−^ hydrogen bonds. Motifs M7, M10, M8 and M9 (TM18), M8 and M9 (TM19), and M7, M10 and M8 (TM20) motifs create the hydrogen-bonded network. This association creates an eight-membered cyclic network with voids of 15 × 9.3 Å which are filled by two phenyl rings of **1** molecules along with the two DMSO molecules that act as guests (see Fig. 10 ▸ *b*).

Stacking of these cyclic networks (viewed along the *c* axis) results in the zigzag hydrogen-bonded sheet structure shown in Fig. 10 ▸(*c*), and these sheets, in turn, stack using van der Waals forces to create the complete three-dimensional crystal structure.

### 3.7. Salt solvate of triamterene with nicotinic acid (**1f**·DMSO)   

The salt obtained by dissolving a 2:1 LAG (DMSO) sample of **1** and **f** crystallizes as a DMSO solvate in the monoclinic space group *P*2_1_/*n* (see Table 1 ▸). The resulting asymmetric unit of the crystal structure is shown in Fig. *S*8 and contains five molecules: two molecules of **1**, one molecule of **f** and two DMSO molecules. In this case, one of the molecules of **1** is protonated (*A*) while the other remains neutral (*B*). A list of hydrogen bonds for **1f**·DMSO is included in Table *S*11.

The crystal structure shows that in the supramolecular tape formed between **1, f** and DMSO, *A* and *B* molecules are connected by M8 motifs in an alternating *AB*–*AB* fashion to produce a basic structural ribbon of **1** molecules to which **f** molecules are attached through the common M7 motif involving N—H⋯O and N^+^—H⋯O^−^ hydrogen bonds. DMSO molecules are connected through M9 motifs, as shown in Fig. 11 ▸(*a*). Motifs M7, M10, M8 and M9 (TM18), M8 and M9 (TM19), and M7, M10 and M8 (TM20) create the hydrogen-bonded tape seen in Fig. 11 ▸(*a*).

Extension of this tape in the second dimension, allows connection between adjacent tapes using weak C—H⋯O hydrogen bonds to produce the hydrogen-bonded sheet shown in Fig. 11 ▸(*b*). Further analysis reveals that the tapes of **1** molecules are stacked in a staggered fashion with the hydro­phobic phenyl groups arranged at a maximum distance from each other (Fig. 12 ▸ *a*). Essentially, *A* molecules interact with the –COO^−^ group of nicotinic acid using the M7 motif and an N—H⋯N hydrogen bond occurs between the N atom of nicotinic acid and the 2-amino group of a *B* molecule with an H⋯N distance of 2.30 Å, as shown in Fig. 12 ▸(*b*).

In the third dimension, molecules are arranged as parallel zigzag stacked sheets, as seen in the space-filling diagram shown in Fig. 13 ▸.

### 3.8. Salt solvate of triamterene with ibuprofen (**1g**·DMSO)   

The salt obtained between **1** and **g** crystallizes as a DMSO solvate in the triclinic space group 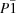, as shown in Table 1 ▸. It is obtained by dissolving a mixture of **1** and **g** in a 2:1 ratio in DMSO. The asymmetric unit is shown in Fig. *S*9 and contains four molecules in total: two molecules of **1** along with one each of **g** and DMSO. As in **1f**·DMSO, one of the molecules of **1** is protonated (*A*), while the other remains neutral (*B*). However, in this case, the DMSO molecule is disordered over two sites with occupancies of 0.9 and 0.1. For the purposes of the crystal structure refinement, geometric constraints and a common isotropic displacement parameter for the non-H atoms were applied to the minor site. A list of hydrogen bonds is included in Table *S*12.

The crystal structure of **1g**·DMSO shows that in the molecular complex the *A* and *B* molecules connect through M4 motifs *via* N—H⋯N hydrogen bonds to yield *AA*–*BB* dimers. These further link through M11 and M12 motifs to create an *AA*–*BB*–*AA* zigzag crinkled tape of **1** molecules (Figs. 14 ▸ and 21 ▸).

The recognition pattern between **1** and **g** shows that N—H⋯N and N—H⋯O^−^ hydrogen bonds through M13 and M14 motifs hold these molecules together. This is one of the most striking differences observed in the crystal structure of **1g**·DMSO in comparison with all adducts described above. In the previous cases, the M7 motif is found between molecules of **1** and all previous coformers containing the –COOH group of **a**–**f**. However, in this case, the M7 motif is absent since molecules of **1** combine to form the M11 motif, as shown in Fig. 15 ▸. At the other end of the combined motif, two molecules of **1** combine with one O atom of the carboxyl group to form the M14 motif. The DMSO molecules interconnect with **1** through the M5 motif, as observed in **1a**·DMSO and **1c**·DMSO. The motifs M5, M4 and M5 (TM16), M13, M4 and M13 (TM21), M11, M12 and M14 (TM22), and M11 and M12 (TM23) make up the hydrogen-bonded tape structure seen in **1g**·DMSO (see Fig. 15 ▸ for details).

Further analysis reveals staggered stacking of the tapes of **1** molecules with the hydro­phobic phenyl groups arranged at a maximum distance from each other (Fig. 16 ▸ *a*). The stacks of **1** connect with each other through **g** molecules involving motifs M13 and M14, which results in the formation of pseudo-cyclic networks (as shown in Fig. 16 ▸ *b* and highlighted in Fig. 16 ▸ *c*).

### 3.9. Analysis of the hydrogen-bonded motifs in 1 and the reported adducts (**1a**–**g**)   

**Figure 17:**
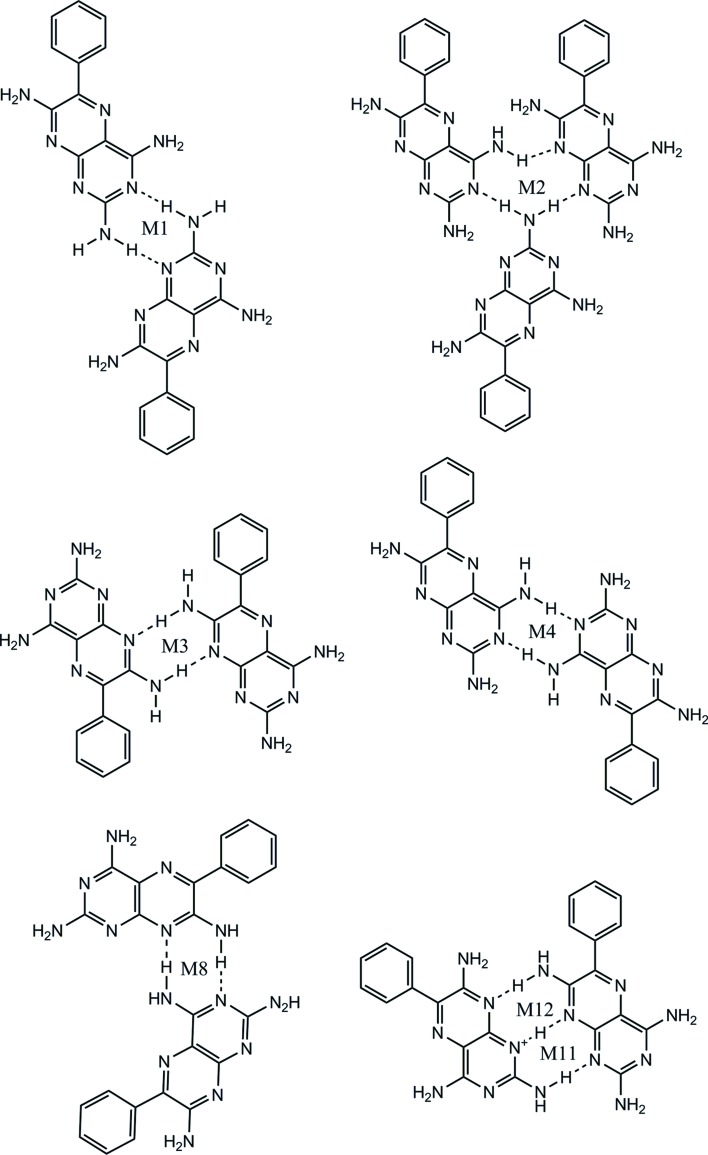
Hydrogen-bonded motifs between molecules of **1** observed in this study.

Overall, 14 different motifs have been observed in **1** and the reported adducts **1a**–**g**·DMSO (see Figs. 17 ▸ and 19). It is recognized that the standard graph-set notation 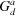
(n) introduced by Etter (1991 ▸) and described in Bernstein *et al.* (1995 ▸) is not sufficient to accurately describe the interactions found in this study, since various ways of forming the same graph set are possible depending upon the specific functional groups involved.

As a result, the concepts of motif and supramolecular synthon have been developed and are used throughout this discussion. Additionally, combined (tape) motifs are identified because of the combination of individual motifs in different structural arrangements.

A survey of the CSD (Version 5.36; *ConQuest* Version 1.18) was conducted in order to better understand the likelihood of these motifs and putative supramolecular synthons occurring in other adducts similar in molecular structure to those found in this study.

#### 3.9.1. Hydrogen-bonded motifs between triamterene (**1**) molecules   

**Figure 18:**
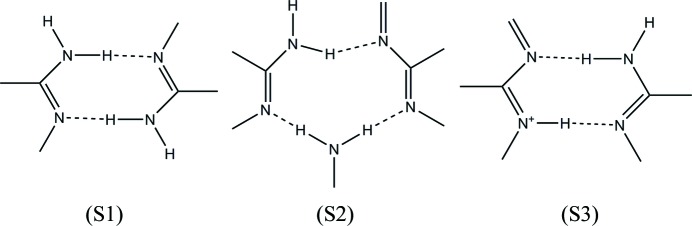
Supramolecular synthons observed in this study.

Molecules of **1** are hydrogen bonded by dimeric N—H⋯N hydrogen bonds between the N atoms at positions 1 and 3 in combination with 2-amino groups creating the M1 motif or supramolecular 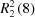 synthon S1 (see Fig. 18 ▸). The M3 motif is created by interaction between the N atoms at position 8 and the 7-amino groups of molecules of **1** on adjacent tapes and may be described as the supramolecular 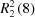 synthon S1. The other homodimeric motifs only found in the salt solvates between molecules of **1** are M4 and M8 and it is clear from Fig. 17 ▸ that they all originate by utilizing different positions of the **1** molecule, but also that they may all be represented by the supramolecular 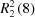 synthon S1.

The M2 motif joins three molecules of **1** utilizing the N atoms at positions 1, 3 and 8, and the 2- and 4-amino groups, and creates the supramolecular 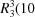) synthon S2 (see Fig. 18 ▸).

The M11 and M12 motifs are unique in this study and exist between two molecules of **1** in **1g**·DMSO, one of which is protonated (*A*) while the other is neutral (*B*). M11 and M12 both possess N^+^—H⋯N and N—H⋯N hydrogen bonds and are represented by the supramolecular 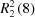 synthon S3 (Fig. 18 ▸).

Although they have the same graph-set descriptor and indeed the same hydrogen bonds, they represent different supramolecular synthons since they utilize different positions on the molecules of **1** involved in their creation.

The seven motifs found between **1** molecules reported here are shown in Fig. 17 ▸.

A CSD analysis undertaken by Delori, Suresh & Pedireddi (2013 ▸) revealed that molecules containing the N1—C1—NH_2_ functionality had a high propensity to form supramolecular synthon S1. In the present study, a search of the CSD shows that, in the solid state, 52.97% (989 out of 1867) of the crystal structures containing this functionality do form supramolecular synthon S1. Indeed, the largest number of motifs (M1, M3, M4 and M8) between molecules of **1** in this study are represented by this supramolecular synthon (see Fig. 18 ▸). Supramolecular synthon S2 is extremely rare and found in only two structures (0.21%) out of the 918 possible crystal structures in the CSD. Finally, the supramolecular synthon S3 is represented by 1.96% (25 structures out of a possible 1277) suitable crystal structures in the CSD.

The various putative supramolecular synthons described in this section are shown in Fig. 18 ▸.

#### 3.9.2. Hydrogen-bonded motifs involving triamterene–solvent (1–solvent) and triamterene–coformer (1–coformer) interactions   

**Figure 19:**
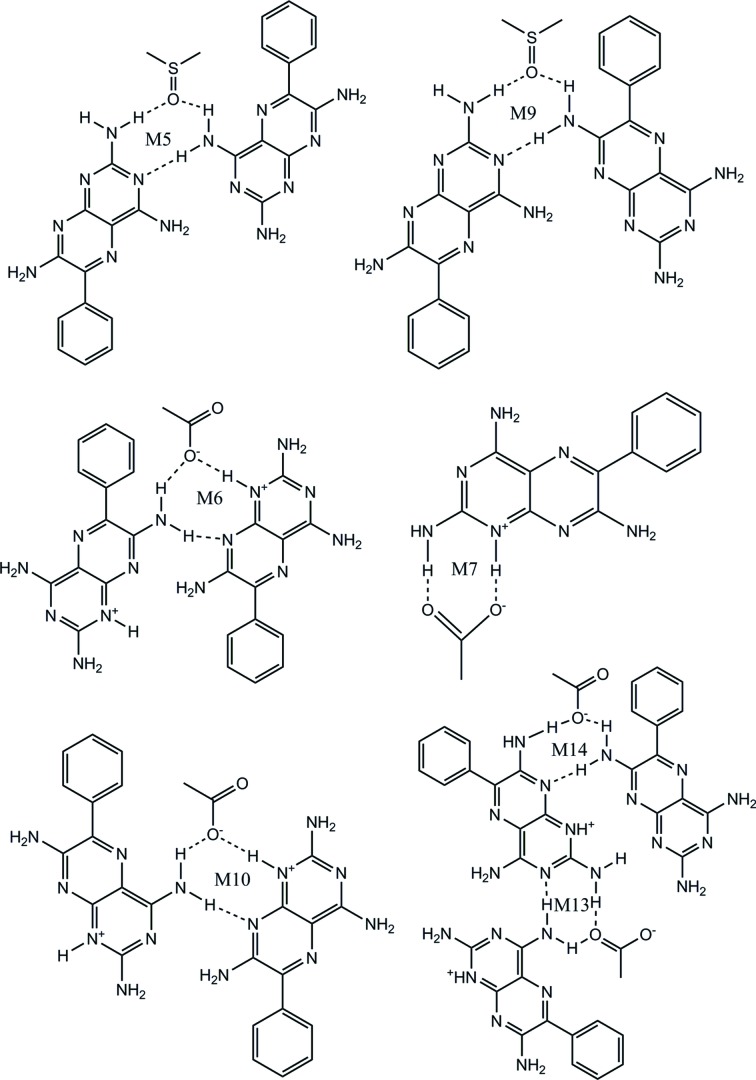
Hydrogen-bonded motifs of **1**–solvent and **1**–coformer molecules observed in this study.

The M5 and M9 motifs occurring between **1** and the solvent (DMSO), and the M6, M7, M10, M13 and M14 motifs resulting from the interaction between **1** and the coformers (**a**–**g**) are shown in Fig. 19 ▸.

**Figure 20:**
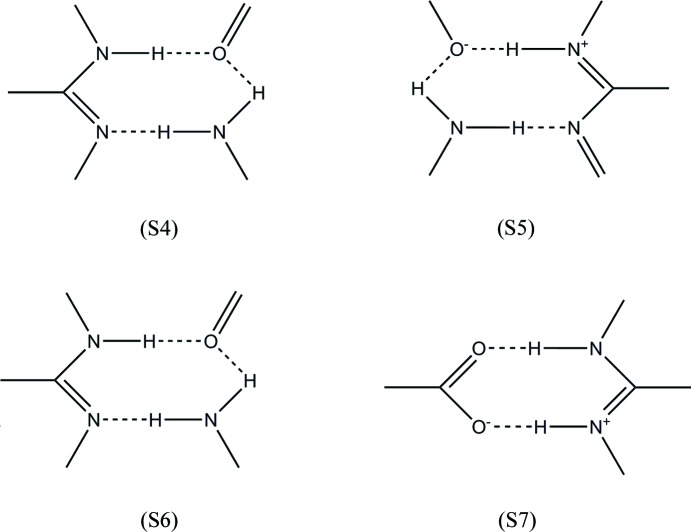
Supramolecular synthons between **1** and solvent, and **1** and coformer molecules observed in this study.

M5 involves the interaction of three molecules including the O atom of DMSO using two N—H⋯O hydrogen bonds with the 2- and 4-amino groups of different molecules of **1** and a further N—H⋯N hydrogen bond between the N3 atom and the 4-amino group of adjacent molecules of **1**. M5 may be represented by the supramolecular 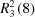 synthon S4 as seen in Fig. 20 ▸ and although M9 is also represented by the supramolecular 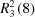 synthon S4, it clearly differs from M5 in the position of one of the amino groups on the molecule of **1**.

The M6 and M10 motifs seen in Fig. 19 ▸ and represented by the supramolecular 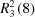 synthon S5 in Fig. 20 ▸ are formed through charge-assisted N^+^—H⋯O^−^and neutral N—H⋯N hydrogen bonds between **1** and the carboxyl group of the acid. Both of these motifs utilize the protonated N atom of **1** at position 1 and the N atom at position 8, but the N—H⋯N hydrogen bond has a different composition in each motif, involving the 7-amino group for M6 and the 4-amino group for M10.

The unique M13 motif or supramolecular 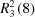 synthon S6 (see Fig. 20 ▸) is only found in **1g**·DMSO. This motif results from an N—H⋯N hydrogen bond between atom N3 and the 4-amino group, along with two N—H⋯O hydrogen bonds involving the 2-amino group of one molecule of **1** and the 4-amino group of the other. Similarly, the unique M14 motif or supramolecular 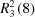 synthon S5 (Fig. 20 ▸) in **1g**·DMSO results from three hydrogen bonds, but in this case, two charge-assisted N—H⋯O^−^ hydrogen bonds and one N—H⋯N hydrogen bond.

In the M7 motif or supramolecular 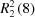 synthon S7 (Fig. 20 ▸) the most basic protonated N atom (N1) and the 2-amino group of **1** interact with the complementary carboxyl group through charge-assisted N^+^—H—O- and N—H⋯O hydrogen bonds. The resulting M7 heterodimer is found to be the most frequent motif seen in this study as it is present in all crystal structures except **1g**·DMSO.

The CSD search carried out as part of this study revealed that supramolecular synthons S4 and S5 have a low probability of occurrence (1.68% each) of all the possible synthons in all structures; supramolecular synthon S6 has a frequency of occurrence of 8.41%, while supramolecular synthon S7 has by far the highest frequency of occurrence at 73.45% (617 out of 840 possible crystal structures). These figures are supported by an earlier CSD study by Allen *et al.* (1999 ▸) that showed the probability of forming the 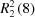 supramolecular synthon as *ca* 95% for mono­carb­oxy­lic acids and 85% for di­carb­oxy­lic acids when no other competing hydrogen-bond donors or acceptors are present. More recently, a study by Delori, Galek *et al.* (2013 ▸) using knowledge-based hydrogen bond propensity calc­ulations (HBPCs) demonstrated the high probability of creating the 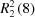 supramolecular synthon when looking at an antimalarial drug,* i.e.* pyrimeth­amine, and various carb­oxy­lic acids.

#### 3.9.3. Hydrogen-bonded tape motifs in triamterene and adducts (1a–g)   

The TM15 tape motif is specific to **1** and is formed by a combination of three motifs, *i.e.* M2, M1 and M2. It is thought the interaction between molecules of **1** to create the M1 motif exposes sites rich in hydrogen-bond donors, *i.e.* the 2-amino groups of two interacting molecules of **1**. The hydrogen-bond acceptor sites on adjacent molecules of **1**, *i.e.* the N1, N3 and N8 atoms, interact with these rich hydrogen-bond donors creating the M2 motif and, therefore, the M15 tape motif with the graph-set notation 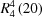. The exposed hydrogen-bond donor sites at the 2- and 4-amino groups also result in the formation of the M4 motif and so can interact with the O atom of DMSO by M5 in adducts **1a** and **1c**·DMSO.

The three motifs (M5, M4 and M5) combine to form the TM16 tape motif with the graph-set notation 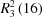. In the TM17 tape motif, the protonated N1 atoms of two molecules of **1** interact with the 2- and 7-amino and the carboxyl groups, resulting in the formation of a tape motif, which is a combination of five motifs (M7, M6, M3, M6 and M7).

In the TM18 tape motif, the exposed hydrogen-bond donor sites that result from M8 at the 2-amino group and the protonated N1 atom are satisfied by the O atom of DMSO and the carboxyl group of the acid to give M9, M10 and M7 motifs. The TM18 tape motif is therefore composed of four motifs (M7, M10, M8 and M9) and may be represented by the graph-set notation 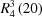. The TM19 tape motif is represented by the graph set notation 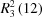 and is a combination of M8 and M9. TM20 resembles TM18 since it contains M7, M10 and M8 in the same order but with the difference that M9 (present in M18) is absent and therefore TM20 may be represented by the graph-set descriptor 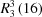.

The TM21 and TM22 tape motifs are unusual since they are present in only one of the adducts (**1g**·DMSO, see Fig. 15 ▸) seen in this study. The TM21 tape motif contains motifs M13, M4 and M13, with donor sites at the 2- and 4-amino groups which are satisfied by an O of the carbonyl group and create the 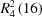 graph set. The TM22 tape motif also contains three motifs (M11, M12 and M14), with donor sites belonging to the 2- and 7-amino groups along with the protonated N1 atom hydrogen bonded to an O atom of the carboxyl group and the N1 atom of the adjacent neutral molecule of **1**.

**Figure 21:**
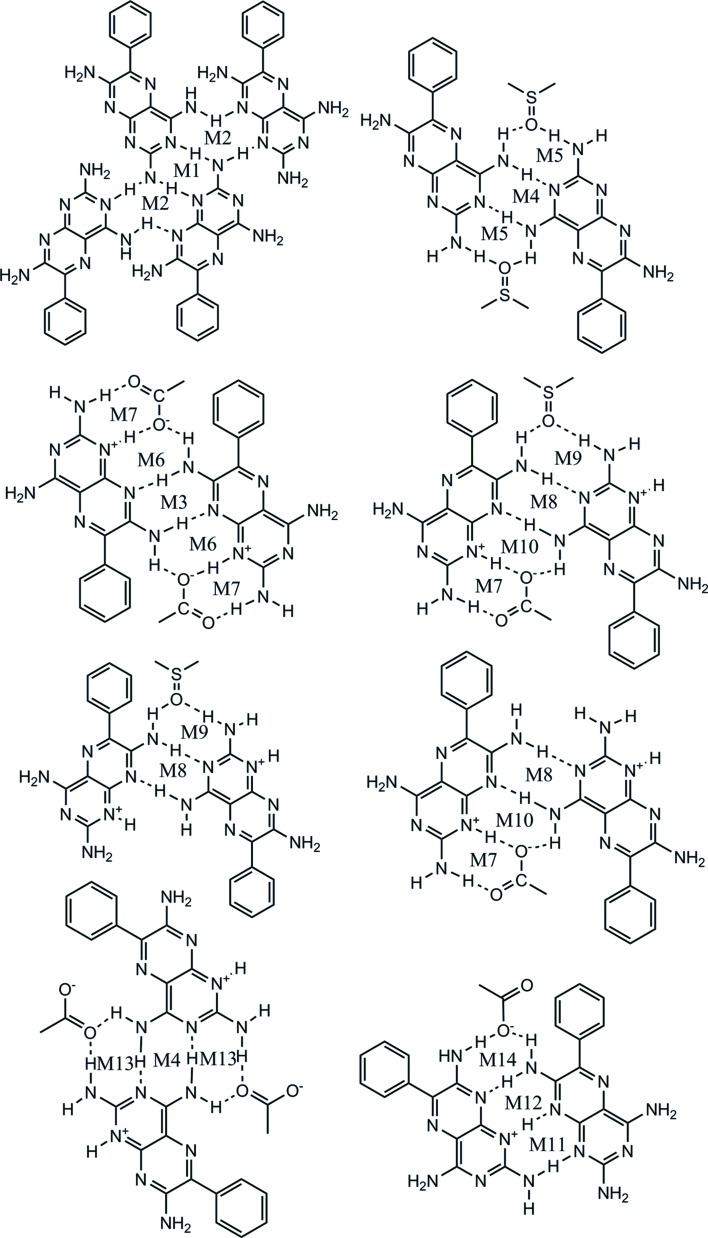
Supramolecular tape motifs found in **1** and adducts of **1** observed in this study.

The combined motifs found in **1** and the adducts of **1** are shown in Fig. 21 ▸.

## 4. Summary   

**Figure 22:**
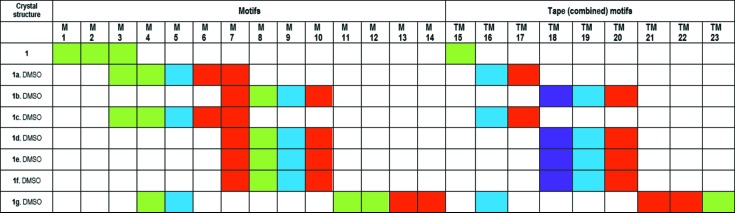
Summary of hydrogen-bond motifs found in this study. Green represents **1**–**1** interactions, blue represents **1**–solvent interactions, red represents **1**–coformer interactions and purple represents solvent–**1**–coformer interactions.

The motifs and tape motifs observed in the hydrogen-bonded ribbons of **1** and the various adducts (**1a**-**g**·DMSO) are summarized in Fig. 22 ▸. By applying colour to represent the motifs, a method of notation (but not mathematical rigour) is developed that better represents the structures. By representing the crystal structure as a repeating sequence of ring structures and by systematically listing the sequence of rings, a pattern is defined. Although this is not the whole story, these patterns can be used to compare the structures.

From Fig. 22 ▸ it can be seen that the crystal structures in this study can be divided into four groups.

(i) **1** can be seen to be composed of only **1**–**1** interactions. The M1 and M2 motifs combine to form the TM15 tape motif and the hydrogen-bonded ribbon. M3 forms an essential connection between adjacent ribbons in **1** that allows the hydrogen-bonded sheet to be formed by extension of the structure in the direction orthogonal to that of the ribbon. Interestingly, M3 is also found in **1a**·DMSO and **1c**·DMSO, but this time within the ribbon structure and it may therefore be regarded as a synthon.

(ii) **1a**·DMSO and **1c**·DMSO contain the motifs M3, M4, M5, M6 and M7 that combine to form TM16 and TM17. These crystal structures contain very similar sheet structures and, looking at the unit-cell dimensions, **1a**·DMSO and **1c**·DMSO may be regarded as being isostructural.

(iii) **1b**·DMSO, **1d**·DMSO, **1e**·DMSO and **1f**·DMSO contain motifs M7, M8, M9 and M10 that combine to form TM18, TM19 and TM20. Interestingly, **1b**·DMSO, **1d**·DMSO and **1e**·DMSO have similar hydrogen-bonded sheets which differ considerably from **1f**·DMSO that contains a hydrogen-bonded double ribbon.

(iv) **1g**·DMSO has a unique connectivity and crystal structure resulting from (in part) the presence of the unique **1**–**1** motifs M11 and M12 that combine to form a unique dimer structure (TM23).

Furthermore, it can be seen from Fig. 22 ▸ that the hydrogen-bonded motif M7 is present in all cases except for **1g**·DMSO. This is one of the motifs predicted by the supramolecular synthon approach and *Δ*p*K*_a_ discussed earlier (see Fig. 2 ▸ for details). The CSD search outlined in §3.9.2 shows the probability of occurrence of M7 in molecules containing the functional groups required to create this motif, such as pyrimethamine (Delori, Galek *et al.*, 2013 ▸) and the triazines (Bhatt *et al.*, 2009 ▸) and carb­oxy­lic acids, is quite high at 73.45%.

To confirm the position of the proton in **1g**·DMSO, a further CSD study was undertaken to look for similar three-centred dimers in the presence of carb­oxy­lic acids. Among the results of this search, the hemicytosinium duplexes described by Perumalla *et al.* (2013 ▸) were found. In this paper, experiments were performed to determine the ability of cytosine to form salts with a series of acids. It was stated that the cytosinium ion was present only with acids of a p*K*_a_ > 4.2 and less strong acids were unable to disrupt the relatively stable duplex structure. It is proposed that a similar kinetic mechanism is found in this study where triamterene forms a stable three-centred duplex (hemitriamterenium) containing the M11 and M12 motifs that cannot be disrupted by ibuprofen with a p*K*_a_ value of 5.2 but can form the triamterenium ion with the stronger carb­oxy­lic acids in the p*K*_a_ range 4.2–4.76.

## 5. Conclusions   

The salt solvate adducts **1a**–**g**·DMSO were prepared using conventional screening techniques, *i.e.* LAG and SEC. Adduct formation was confirmed by SCXRD, PXRD, DSC and TGA. The results are in accordance with the modified Δp*K*_a_ rule proposed by Cruz-Cabeza (2012 ▸). To overcome some of the limitations of graph-set theory, the concepts of motif, tape motif and supramolecular synthon are introduced to describe the crystal structures since it has been established that molecules of **1** can adopt different orientations corresponding to the same graph-set descriptor. It is noteworthy that the M1 motif is seen only in the crystal structure of **1** but is not observed in any of the other crystal structures because of its replacement by M7 in **1a**–**f**·DMSO and M11 in **1g**·DMSO. The present study and the CSD survey carried out by Delori, Suresh & Pedireddi (2013 ▸) both suggest M7 to be a very robust motif. In fact, the M7 motif is present in all the reported adducts found in this study except for **1g**·DMSO. It should also be noted that this is the same motif proposed in Fig. 2 ▸, resulting from the crystal engineering approach which emphasizes the identification of systematic patterns of hydrogen bonds from known crystal structures. Overall seven adducts (**1a**–**g**. DMSO) containing 14 motifs, 8 supramolecular tape motifs and 7 supramolecular synthons are reported and their structural relationships explored. The anomalous behaviour of **1 g**·DMSO is explained by the inability of ibuprofen to disrupt the stable hemitriamterenium duplex.

## Supplementary Material

Crystal structure: contains datablock(s) 1a_DMSO, 1b_DMSO, 1c_DMSO, 1d_DMSO, 1e_DMSO, 1f_DMSO, 1g_DMSO. DOI: 10.1107/S2052252518003317/lq5012sup1.cif

Structure factors: contains datablock(s) 1a_DMSO. DOI: 10.1107/S2052252518003317/lq50121a_DMSOsup2.hkl

Structure factors: contains datablock(s) 1b_DMSO. DOI: 10.1107/S2052252518003317/lq50121b_DMSOsup3.hkl

Structure factors: contains datablock(s) 1c_DMSO. DOI: 10.1107/S2052252518003317/lq50121c_DMSOsup4.hkl

Structure factors: contains datablock(s) 1d_DMSO. DOI: 10.1107/S2052252518003317/lq50121d_DMSOsup5.hkl

Structure factors: contains datablock(s) 1e_DMSO. DOI: 10.1107/S2052252518003317/lq50121e_DMSOsup6.hkl

Structure factors: contains datablock(s) 1f_DMSO. DOI: 10.1107/S2052252518003317/lq50121f_DMSOsup7.hkl

Structure factors: contains datablock(s) 1g_DMSO. DOI: 10.1107/S2052252518003317/lq50121g_DMSOsup8.hkl

Further details of experimental work. DOI: 10.1107/S2052252518003317/lq5012sup9.pdf

Enhanced 3D images in MERCURY format. DOI: 10.1107/S2052252518003317/lq5012sup10.zip

CCDC references: 1579683, 1579684, 1579685, 1579686, 1579687, 1579688, 1579689

## Figures and Tables

**Table 1:** Selected crystallographic parameters for **1** and **1a**–**g**·DMSO

	**1** †	**1a**·DMSO	**1b**·DMSO	**1c**·DMSO	**1d**·DMSO	**1e**·DMSO	**1f**·DMSO	**1g**·DMSO
Formula	C_12_H_11_N_7_	C_12_H_12_N_7_^+^·C_2_H_3_O_2_^−^·C_2_H_6_OS	C_12_H_12_N_7_^+^·C_4_H_5_O_4_^−^·C_2_H_6_OS	2C_12_H_12_N_7_^+^·C_6_H_8_O_4_^2−^·2C_2_H_6_OS	2C_12_H_12_N_7_^+^·C_7_H_10_O_4_^2−^·2C_2_H_6_OS	2C_12_H_12_N_7_^+^·C_9_H_14_O_4_^2−^·2C_2_H_6_OS	C_12_H_12_N_7_^+^·C_6_H_4_NO_2_^−^·C_12_H_11_N_7_.2C_2_H_6_OS	C_12_H_12_N_7_^+^·C_13_H_17_O_2_^−^·C_12_H_11_N_7_·C_2_H_6_OS
*M*_r_	253.28	391.46	449.49	808.95	822.98	851.03	785.92	790.96
Crystal system	Triclinic	Triclinic	Monoclinic	Triclinic	Triclinic	Monoclinic	Monoclinic	Triclinic
Space group	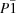	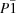	*P*2_1_/*c*	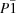	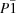	*P*2_1_/*n*	*P*2_1_/*n*	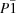
*a* (Å)	7.4432 (15)	10.8022 (2)	13.5226 (7)	11.0286 (3)	14.0750 (5)	15.0338 (3)	14.8461 (2)	11.2398 (3)
*b* (Å)	9.993 (2)	13.9084 (2)	14.9966 (7)	13.7429 (4)	14.8889 (6)	11.5004 (2)	12.3081 (3)	13.2439 (3)
*c* (Å)	16.648 (3)	14.6076 (3)	10.6958 (5)	14.9742 (5)	19.635 (2)	24.3960 (6)	21.2850 (5)	15.4624 (4)
α (°)	77.55 (2)	115.284 (1)	90	115.247 (2)	86.408 (2)	90	90	113.595 (2)
β (°)	87.54 (3)	109.088 (2)	104.179 (2)	109.951 (2)	88.142 (2)	104.539 (1)	105.176 (1)	103.527 (2)
γ (°)	87.09 (3)	90.525 (1)	90	90.122 (2)	71.702 (2)	90	90	91.977 (2)
*V* (Å^−3^)	1207.0 (4)	1846.64 (6)	2102.95 (18)	1900.24 (11)	3898.7 (5)	4082.87 (15)	3753.71 (14)	2029.46 (9)
*Z*	4	4	4	2	4	4	4	2
*D*_calc_ (Mg m^−1^)	1.394	1.408	1.420	1.414	1.402	1.384	1.391	1.294
*T* (K)	180 (2)	180 (2)	220 (2)	180 (2)	150 (2)	180 (2)	180 (2)	180 (2)
GOF on *F* ^2^	1.032	0.935	0.747	1.022	0.945	1.033	0.897	1.021
*R*_1_ [*I* > 2*σ*(*I*)]	0.0360	0.0466	0.0364	0.0729	0.0788	0.0587	0.0414	0.0737
w*R*_2_	0.0916	0.1179	0.0693	0.1727	0.1951	0.1461	0.0803	0.1891
CCDC	1532364	1579683	1579684	1579685	1579686	1579687	1579688	1579689

† The values for **1** were obtained from the work by Hughes *et al.* (2017 ▸).
