# Supplementary material for: Structural studies of crystalline forms of triamterene with carboxylic acid, GRAS and API molecules
Source: IUCrJ. 2018 Apr 6;5(Pt 3):309–24. doi: 10.1107/S2052252518003317 (PMC5929377; doi:10.1107/S2052252518003317)
Supplement: Supplementary file 9 [file m-05-00309-sup9.pdf]

IUCrJ

Volume 5 (2018)

Supporting information for article:

Structural studies of crystalline forms of triamterene with carboxylic acid, GRAS and API molecules

Abida Rehman, Amit Delori, David S. Hughes and William Jones

S1. Triamterene and coformers used in this study

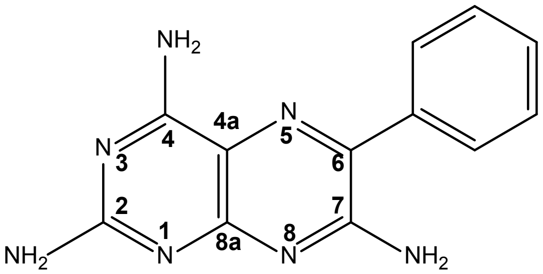

Triamterene (**1**)

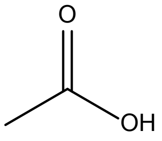

Acetic acid (**a**)

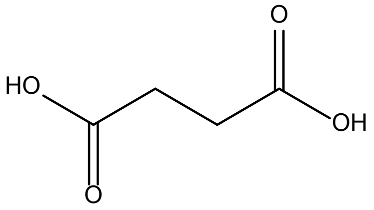

Succinic acid (**b**)

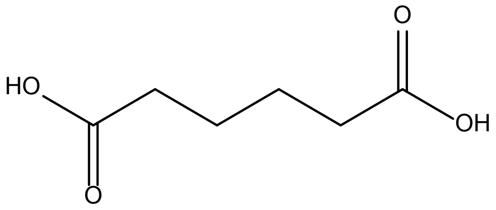

Adipic acid (**c**)

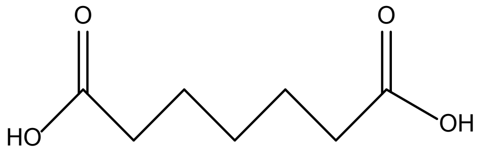

Pimelic acid (**d**)

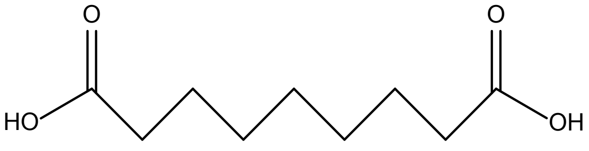

Azelaic acid (**e**)

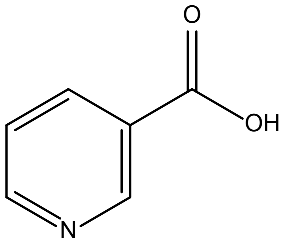

Nicotinic acid (**f**)

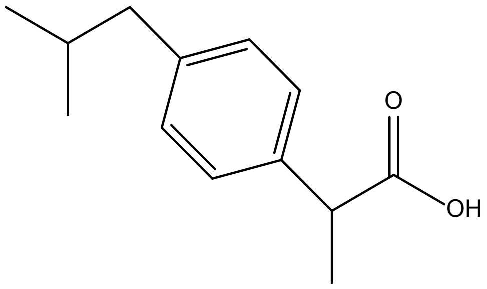

Ibuprofen (**g**)

Figure S1 Triamterene (**1**) and coformers (**a–g**) considered in the present study.

S2. Summary of the adduct prediction data and results obtained by experiment

S2.1. Method

Predictions of the adduct formation reactions considered in this study involving **1** and coformers (**a**–**g**) in DMSO are expressed as P_{obs} which is the probability of observing *AB* (cocrystal) and $A^{-}B^{+}$ (salt) in % terms according to the Cruz-Cabeza (2012) formula.

P_{obs} $(AB, \%) = -17\ \Delta pK_a + 72$ for $-1 \leq \Delta pK_a \leq 4$

P_{obs} $(A^{-}\ B^{+}, \%) = 17\ \Delta pK_a + 28$ for $-1 \leq \Delta pK_a \leq 4$

Where P_{obs} is the probability of observing the *A-B* (cocrystal) and $A^{-}\ B^{+}$ (salt) in % terms.

Using this method the % probability (P_{obs}) values for the proposed reactions were calculated and compared with the experimental results and summarized in Table S1.

S2.2. Results

Table S1 Results of the adduct formation reactions considered in this study.

Acid	Base	P_{obs} (*AB*, %)	P_{obs} ($A^{-}B^{+}$, %)	ΔpK_a	Predicted result	Experimental result	Product
**a**	**1**	52.1	47.9	1.17	cocrystal	salt	**1a**·DMSO
**b**	**1**	42.4	57.6	1.74	salt	salt	**1b**·DMSO
**c**	**1**	46.3	53.7	1.51	salt	salt	**1c**·DMSO
**d**	**1**	47.3	52.7	1.45	salt	salt	**1d**·DMSO
**e**	**1**	48.5	51.5	1.38	salt	salt	**1e**·DMSO
**f**	**1**	45.0	55.0	1.59	salt	salt	**1f**·DMSO
**g**	**1**	54.7	43.3	1.02	cocrystal	salt	**1g**·DMSO

Notes:

- Δp*Ka* is the difference in the p*Ka* of the most basic atom on **1** and the most acidic atom on the complementary acid. p*Ka* values of **1** are calculated using the SPARC online calculator, http://sparc.chem.uga.edu/sparc while p*Ka* values of the coformers (**a–f**) are obtained from data compiled by R. Williams, http://research.chem.psu.edu/brpgroup/pKa_compilation.pdf.
- For ibuprofen (**g**) the p*Ka* data is obtained from Sangster J; LOGKOW Databank, Sangster Res. Lab., Montreal, Quebec, Canada (1994).
- Predicted and experimental results of the adduct formation reactions refer to the form predicted according to the Cruz-Cabeza formula and the actual form obtained by crystallization.

S3. Summary of crystallization data

Table S2 Synthesis, crystallization experiment and subsequent product designation used throughout this study

	Triamterene (mg/mmol)	Coformer (mg/mmol)	Triamterene: Coformer Ratio	Crystallization Solvent (ml)	Time to crystallization (days)	Product designation
**Triamterene**	10/0.039	N/A	N/A	MeOH (30)	7	**1**
**Triamterene, acetic acid and DMSO**	40.42/0.159	9.58/0.159	1 : 1	DMSO (2)	4	**1a**·DMSO
**Triamterene, succinic acid and DMSO**	34.1/0.135	15.9/0.135	1 : 1	DMSO (2)	3	**1b**·DMSO
**Triamterene, adipic acid and DMSO**	38.80/0.077	11.20/0.077	2 : 1	DMSO (2)	4	**1c**·DMSO
**Triamterene, pimelic acid and DMSO**	37.99/0.075	12.01/0.075	2 : 1	DMSO (2)	5	**1d**·DMSO
**Triamterene, azelaic acid and DMSO**	36.45/0.072	13.55/0.072	2 : 1	DMSO (2)	5	**1e**·DMSO
**Triamterene, nicotinic acid and DMSO**	40.22/0.079	9.78/0.079	2 : 1	DMSO (2)	4	**1f**·DMSO
**Triamterene, ibuprofen and DMSO**	35.53/0.070	14.47/0.070	2 : 1	DMSO (2)	6	**1g**·DMSO

S4. Single crystal data

Table S3 Crystallographic parameters for **1** and the salt solvates (**1a**–**g**·DMSO).

	**1**	**1a·DMSO**	**1b·DMSO**	**1c·DMSO**	**1d·DMSO**	**1e·DMSO**	**1f·DMSO**	**1g·DMSO**
**Formula**	$C_{12}H_{11}N_7$	$[C_{12}H_{12}N_7]^+$ $[C_2H_3O_2]^-$. C_2H_6OS	$[C_{12}H_{12}N_7]^+$ $[C_4H_5O_4]^-$. C_2H_6OS	$2[C_{12}H_{12}N_7]^+$ $[C_6H_8O_4]^{2-}$. $2C_2H_6OS$	$2[C_{12}H_{12}N_7]^+$ $[C_7H_{10}O_4]^{2-}$. $2C_2H_6OS$	$2[C_{12}H_{12}N_7]^+$ $[C_9H_{14}O_4]^{2-}$. $2C_2H_6OS$	$[C_{12}H_{12}N_7]^+$ $[C_6H_4NO_2]^-$. $C_{12}H_{11}N_7$. $2C_2H_6OS$	$[C_{12}H_{12}N_7]^+$ $[C_{13}H_{17}O_2]^-$. $C_{12}H_{11}N_7$. C_2H_6OS
**Mr**	253.28	391.46	449.49	808.95	822.98	851.03	785.92	790.96
**Crystal habit**	block	block	block	block	block	block	needle	block
**Crystal colour**	yellow	colourless	colourless	colourless	colourless	colourless	colourless	colourless
**Crystal system**	triclinic	triclinic	monoclinic	triclinic	triclinic	monoclinic	monoclinic	triclinic
**Space group**	$P\bar{1}$	$P\bar{1}$	$P2_1/c$	$P\bar{1}$	$P\bar{1}$	$P2_1/n$	$P2_1/n$	$P\bar{1}$
a **(Å)**	7.4432(15)	10.8022(2)	13.5226(7)	11.0286(3)	14.0750(5)	15.0338(3)	14.8461(2)	11.2398(3)
b **(Å)**	9.993(2)	13.9084(2)	14.9966(7)	13.7429(4)	14.8889(6)	11.5004(2)	12.3081(3)	13.2439(3)
c **(Å)**	16.648(3)	14.6076(3)	10.6958(5)	14.9742(5)	19.635(2)	24.3960(6)	21.2850(5)	15.4624(4)
**α (°)**	77.55(2)	115.284(1)	90	115.247(2)	86.408(2)	90	90	113.595(2)
**β (°)**	87.54(3)	109.088(2)	104.179(2)	109.951(2)	88.142(2)	104.539(1)	105.176(1)	103.527(2)
**γ (°)**	87.09(3)	90.525(1)	90	90.122(2)	71.702(2)	90	90	91.977(2)
V **($Å^{-3}$)**	1207.0(4)	1846.64(6)	2102.95(18)	1900.24(11)	3898.7(5)	4082.87(15)	3753.71(14)	2029.46(9)
Z	4	4	4	2	4	4	4	2
**D_{calc} (gcm^{-1})**	1.394	1.408	1.420	1.414	1.402	1.384	1.391	1.294
T**(K)**	180(2)	180(2)	220(2)	180(2)	150(2)	180(2)	180(2)	180(2)
**λKα (Cu or Mo source)**	1.54178 (Cu)	0.7107 (Mo)	0.71073 (Mo)	0.71073 (Mo)	0.71073 (Mo)	0.71070 (Mo)	0.71070 (Mo)	0.71070 (Mo)
**μ (mm^{-1})**	0.761	0.209	0.200	0.205	0.202	0.195	0.203	0.136
**2θ range (°)**	67.679	52.924	47.790	45.998	43.762	43.632	47.742	50.262
**Limiting indices**	$-8 \le h \le 9$ $-12 \le k \le 12$ $-20 \le l \le 20$	$-15 \le h \le 14$ $-19 \le k \le 18$ $-14 \le l \le 20$	$-14 \le h \le 17$ $-19 \le k \le 19$ $-13 \le l \le 13$	$-13 \le h \le 12$ $-16 \le k \le 16$ $-18 \le l \le 18$	$-16 \le h \le 16$ $-17 \le k \le 17$ $-19 \le l \le 23$	$-18 \le h \le 17$ $-13 \le k \le 13$ $-27 \le l \le 29$	$-18 \le h \le 18$ $-15 \le k \le 15$ $-23 \le l \le 27$	$-14 \le h \le 14$ $-17 \le k \le 16$ $-19 \le l \le 20$
F **(000)**	528	824	944	852	1736	1800	1648	836
**No. of reflections measured**	31347	24626	12060	25204	32837	22149	23512	30734
**No. unique reflns.**	4571	10617	4586	6951	13988	7406	8467	9204
**No. of reflns. used**	3786	6401	2099	5118	5187	4611	4823	6189
**No. of parameters**	432	655	372	520	1035	705	652	626
**GOF on F^2**	1.032	0.935	0.747	1.022	0.945	1.033	0.897	1.021
**R_1 [$I>2\sigma(I)$]**	0.0360	0.0466	0.0364	0.0729	0.0788	0.0587	0.0414	0.0737
**wR_2**	0.0916	0.1179	0.0693	0.1727	0.1951	0.1461	0.0803	0.1891
**Final diff. Fourier map (e^- $Å^{-3}$) max., min.**	0.248 and -0.187	0.673 and -0.442	0.309 and -0.306	1.614 and -0.664	0.775 and -0.377	1.258 and -0.399	0.248 and -0.381	1.213 and -0.417
**CCDC deposition number**	1532364	1579683	1579684	1579685	1579686	1579687	1579688	1579689
**Reference**	(Hughes et al., 2017)	This work	This work	This work	This work	This work	This work	This work

S5. ORTEP diagrams and hydrogen bond tables

All asymmetric units are drawn using *ORTEP-3 for Windows* (Farruga, 2012) and employ a labelling scheme consistent with IUPAC and IUCr recommendations for small molecules (see Table S4).

Table S4 Crystallographic labelling of molecules of **1** and **1a–g** and DMSO.

	**Triamterene (1)**	**Coformer (a–g)**	**Solvent (DMSO)**
**1**	A, B	None	None
**1a**·DMSO	A, B	E, F	C, D
**1b**·DMSO	A	B	C
**1c**·DMSO	A, B	[0.5]C, [0.5]D	E, F
**1d**·DMSO	A, B, C, D	E, F	G, H, I , J
**1e**·DMSO	A, B	C	D, E
**1f**·DMSO	A, B	C	D, E
**1g**·DMSO	A, B	C	D

The hydrogen bond tables that follow (Tables S5 – S12) were produced using *PLATON* (Spek, 2009) and contain details of D-H···A bonds and angles generated for hydrogen bonds satisfying the default criteria of distance (D···A) being < R(D) + R(A) + 0.50Å whilst that of (H···A) is < R(H) + R(A) - 0.12 Å and angle (D-H···A) is > 100.00°; where D is a potential donor, A is a potential acceptor and R is the radius of the designated atom type. In cases where it is obvious that the directed hydrogen bond contributes to the formation of the hydrogen bonded sheet (but is slightly longer than expected) the default criteria have been relaxed and the resulting contacts are highlighted in red (see Tables S6, S7 and S11).

S5.1. Triamterene (1)

Crystallographic data for **1** is taken from Hughes *et al*. (2017) and renumbered for the systematic purposes of this study.

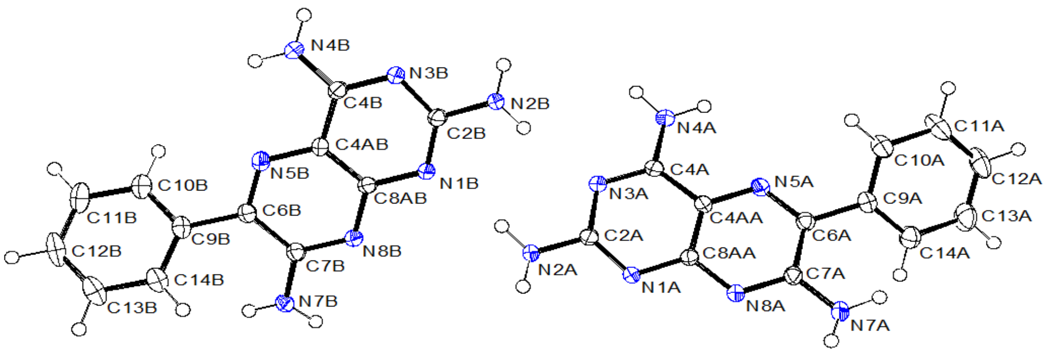

Figure S2 *ORTEP* diagram drawn at 50% probability for **1**.

Table S5 Hydrogen bond table for **1**.

No.	Type	Res	Donor--H...A	[ARU]	D--H	H...A	D...A	D--H...A
1		1	N2A—H2A…N3B	[1655.02]	0.887(15)	2.167(15)	3.0430(17)	169.4(16)
2		2	N2B—H2B…N3A	[1555.01]	0.920(16)	2.161(15)	3.0682(17)	168.6(15)
3		1	N2A—H3A…N1B	[1555.02]	0.922(15)	2.141(15)	3.0583(16)	173.1(14)
4		2	N2B—H3B…N1A	[1455.01]	0.911(15)	2.138(15)	3.0436(16)	172.7(14)
5		1	N4A—H4A…N8A	[1455.01]	0.92(2)	2.43(2)	3.1159(17)	131.3(15)
6		2	N4B—H4B…N8B	[1455.02]	0.90(2)	2.46(2)	3.1130(17)	130.4(14)
7	INTRA	1	N4A—H5A…N5A	[ ]	0.921(18)	2.399(15)	2.7668(16)	103.7(11)
8		1	N4A—H5A…N7A	[1455.01]	0.921(18)	2.597(16)	3.1791(18)	121.7(12)
9	INTRA	2	N4B—H5B…N5B	[ ]	0.916(18)	2.412(15)	2.7762(17)	103.7(11)
10		1	N7A—H6A…N2B	[2767.02]	0.909(18)	2.338(17)	3.0426(17)	134.3(14)
11		2	N7B—H6B…N2A	[2776.01]	0.889(18)	2.323(18)	3.0323(17)	136.7(14)
12		1	N7A—H7A…N8A	[2867.01]	0.905(16)	2.146(16)	3.0473(17)	173.5(15)
13		2	N7B—H7B…N8B	[2776.02]	0.913(16)	2.125(16)	3.0288(17)	170.1(15)
14	INTRA	1	C14A—H14A…N7A	[ ]	0.974(15)	2.597(16)	3.0149(19)	106.0(11)
15	INTRA	2	C14B—H14B…N7B	[ ]	0.973(15)	2.544(15)	2.9913(19)	108.1(11)

Translation of ARU-code to CIF and Equivalent Position Code:

[1655.] = [1_655] = 1+x, y, z

[2776.] = [2_776] = 2-x, 2-y, 1-z

[1455.] = [1_455] = -1+x, y, z

[2767.] = [2_767] = 2-x, 1-y, 2-z

[2867.] = [2_867] = 3-x, 1-y, 2-z

S5.2. Triamterene and acetic acid (1a·DMSO)

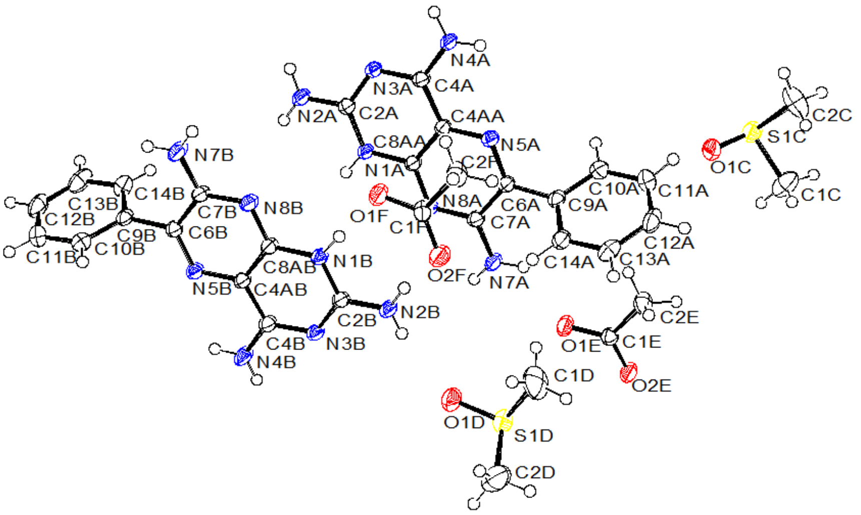

Figure S3 *ORTEP* diagram drawn at 50% probability for **1a**·DMSO.

Table S6 Hydrogen bond table for **1a**·DMSO.

No.	Type	Res	Donor--H...A	[ARU]	D--H	H...A	D...A	D--H...A
1		1	N1A—H1XA…O1E	[2666.05]	0.91(2)	1.82(2)	2.7257(19)	175.7(17)
2		2	N1B—H1XB…O1F	[1555.06]	0.89(2)	1.84(2)	2.7278(19)	176.4(17)
3		1	N2A—H2A…O2E	[2666.05]	0.87(2)	1.87(2)	2.735(2)	174(2)
4		2	N2B—H2B…O2F	[1555.06]	0.90(2)	1.83(2)	2.715(2)	169(2)
5		1	N2A—H3A…O1C	[2566.03]	0.84(2)	2.10(2)	2.906(2)	162(2)
6		2	N2B—H3B…O1D	[1555.04]	0.803(19)	2.28(2)	3.065(2)	168(2)
7		1	N4A—H4A…N3B	[1455.02]	0.87(2)	2.15(2)	3.017(2)	172.2(19)
8		2	N4B—H4B…N3A	[1655.01]	0.81(2)	2.20(2)	3.009(2)	177(2)
9		1	N4A—H5A…O1D	[1455.04]	0.85(2)	2.09(2)	2.807(2)	141.9(17)
10	INTRA	1	N4A—H5A…N5A	[ ]	0.85(2)	2.526(19)	2.807(2)	100.5(14)
11	INTRA	2	N4B—H5B…N5B	[ ]	0.86(3)	2.47(2)	2.787(2)	102.9(16)
12		2	N4B—H5B…O1C	[2666.03]	0.86(3)	2.22(2)	2.883(2)	134.4(18)
13		1	N7A—H6A…O1E	[1555.05]	0.83(2)	2.165(18)	2.768(2)	129.3(16)
14		2	N7B—H6B…O1F	[2565.06]	0.82(2)	2.246(19)	2.833(2)	128.7(16)
15		1	N7A—H7A…N8A	[2666.01]	0.91(2)	2.20(2)	3.102(2)	170(2)
16		2	N7B—H7B…N8B	[2565.02]	0.87(2)	2.23(2)	3.089(2)	172(2)
17		1	C10A—H10A…N3A	[2566.01]	0.925(19)	2.579(19)	3.482(2)	165.8(15)
18		1	C14A—H14A…O2F	[1555.06]	0.94(2)	2.51(2)	3.315(2)	144.4(16)
19		2	C14B—H14B…O2E	[2666.05]	0.90(2)	2.57(2)	3.372(2)	150(2)
20		1	C13A-H13A…O2E	[2656.05]	0.84(2)	2.64(2)	3.347(3)	142.5(18)
21		2	C13B-H13B…O2F	[1565.06]	0.94(2)	2.61(3)	3.464(3)	156.3(19)

Translation of ARU-code to CIF and Equivalent Position Code:

[2666.] = [2_666] = 1-x, 1-y, 1-z

[2566.] = [2_566] = -x, 1-y, 1-z

[1455.] = [1_455] = -1+x, y, z

[1655.] = [1_655] = 1+x, y, z

[2565.] = [2_565] = -x, 1-y, -z

[2656.] = [2_656] = 1-x, -y, 1-z

[1565.] = [1_565] = x, 1+y, z

S5.3. Triamterene and succinic acid (1b·DMSO)

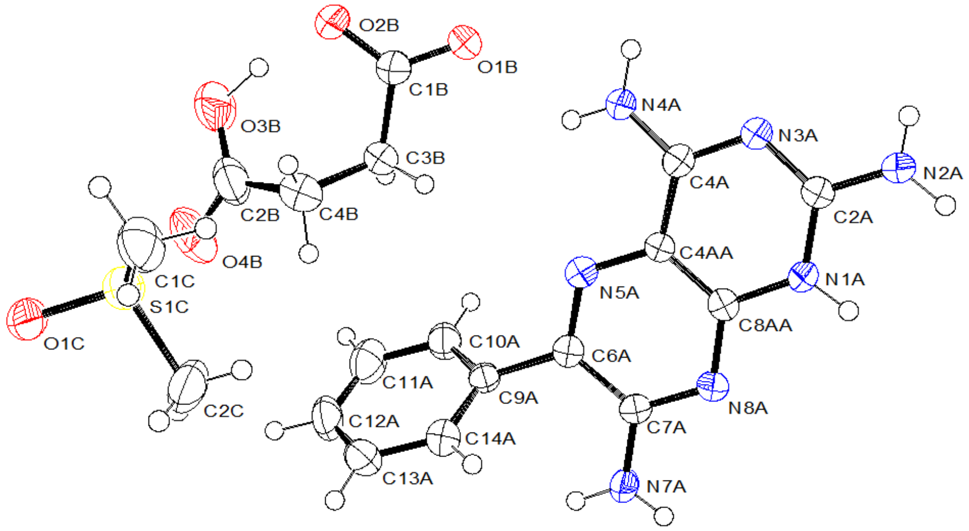

Figure S4 *ORTEP* diagram drawn at 50% probability for **1b**·DMSO.

Table S7 Hydrogen bond table for **1b**·DMSO.

No.	Type	Res	Donor--H…A	[ARU]	D--H	H…A	D…A	D--H…A
1		1	N1A—H1XA…O1B	[2746.02]	0.889(15)	1.866(15)	2.7555(17)	179.0(17)
2		1	N2A—H2A…O2B	[2746.02]	0.915(16)	1.938(16)	2.843(2)	169.4(14)
3		1	N2A—H3A…O1C	[1656.03]	0.816(15)	2.122(15)	2.924(2)	167.7(14)
4	INTRA	2	O3B—H3B…O2B	[ ]	1.05(2)	1.49(2)	2.526(2)	169(2)
5		1	N4A—H4A…N8A	[2756.01]	0.902(19)	2.192(19)	3.088(2)	172.1(17)
6		1	N4A—H5A…O1B	[1555.02]	0.873(17)	2.192(15)	2.810(2)	127.5(12)
7	INTRA	1	N4A—H5A…N5A	[ ]	0.873(17)	2.492(14)	2.807(2)	102.1(10)
8		1	N7A—H6A…O1C	[2645.03]	0.885(15)	2.076(14)	2.777(2)	135.5(12)
9		1	N7A—H7A…N3A	[2746.01]	0.817(15)	2.222(15)	3.031(2)	170.5(15)
10		3	C1C—H2C…O4B	[4555.02]	0.96(2)	2.35(2)	3.213(3)	150.7(15)
11		3	C2C—H6C…O4B	[4555.02]	0.85(2)	2.616(19)	3.385(4)	150.5(15)

Translation of ARU-code to CIF and Equivalent Position Code:

[2746.] = [2_746] = 2-x, -½+y, 3/2-z

[4555.] = [4_555] = -x, ½-y, ½+z

[1656.] = [1_656] = 1+x, y, 1+z

[2756.] = [2_756] = 2-x, ½+y, 3/2-z

[2645.] = [2_645] = 1-x, -1/2+y, ½-z

S5.4. Triamterene and adipic acid (1c·DMSO)

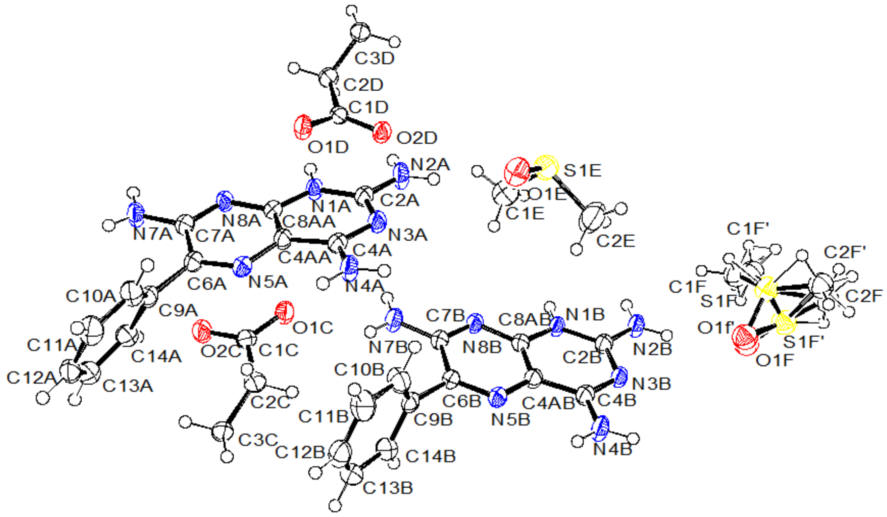

Figure S5 *ORTEP* diagram drawn at 50% probability for **1c**·DMSO.

Table S8 Hydrogen bond table for **1c**·DMSO.

No.	Type	Res	Donor--H…A	[ARU]	D--H	H…A	D…A	D--H…A
1		1	N2A—H2A…O2D	[1555.04]	0.88	1.90	2.772(4)	173
2		2	N2B—H2B…O2C	[2666.03]	0.88	1.91	2.748(4)	157
3		1	N2A—H3A…O1E	[1555.05]	0.88	2.18	3.048(5)	168
4		2	N2B—H3B…O1F	[1555.06]	0.88	2.18	3.040(8)	167
5		1	N4A—H4A…N3B	[2566.02]	0.88	2.13	2.996(5)	167
6		2	N4B—H4B…N3A	[2566.01]	0.88	2.15	3.021(5)	169
7	INTRA	1	N4A—H5A…N5A	[ ]	0.88	2.46	2.788(5)	102
8		1	N4A—H5A…O1F	[2566.06]	0.88	2.08	2.808(7)	140’
9	INTRA	2	N4B—H5B…N5B	[ ]	0.88	2.45	2.773(5)	103
10		2	N4B—H5B…O1E	[2566.05]	0.88	2.21	2.889(5)	133’
11		1	N7A—H6A…O1D	[2655.04]	0.84(4)	2.21(4)	2.832(5)	131(4)
12		2	N7B—H6B…O1C	[1555.03]	0.84(4)	2.33(4)	2.911(5)	128(4)
13		1	N1A—H1XA…O1D	[1555.04]	0.83(5)	1.85(5)	2.681(5)	178(4)
14		1	N7A—H7A…N8A	[2655.01]	0.82(5)	2.27(5)	3.073(5)	169(5)
15		2	N7B---H7B…N8B	[2666.02]	0.87(6)	2.24(6)	3.101(5)	170(5)
16		2	N1B—H1XB…O1C	[2666.03]	0.93(6)	1.77(6)	2.687(5)	170(5)
17		6>	C1F---H1F…O2C	[2666.03]	0.98	2.53	3.500(9)	170
18		6>	C1F—H2F…O1D	[1566.04]	0.98	2.51	3.489(8)	175
19		5	C1E—H3E…O1C	[2666.03]	0.98	2.56	3.319(5)	135
20		1	C10A—H10A…N3A	[2555.01]	0.95	2.50	3.444(5)	172
21		2	C11B—H11B…S1F	[2566.06]	0.95	2.82	3.641(5)	146
22		1	C13A---H13A…O2D	[1545.04]	0.95	2.55	3.293(5)	135

Translation of ARU-code to CIF and Equivalent Position Code:

[2566.] = [2_566] = -x, 1-y, 1-z

[2555.] = [2_555} = -x, -y, -z

[2655.] = [2_655] = 1-x, -y, -z

[1545.] = [1_545} = -x, -1+y, z

[2666.] = [2_666] = 1-x, 1-y, 1 -z

[1566.] = [1_566] = x, 1+y, 1+z

[2566.] = [2_566] = -x, 1-y, 1-z

[2666.] = [2_666] = 1-x, 1-y, 1-z

[2556.] = [2_556] = -x, -y, 1-z

[1556.] = [1_556] = x, y, 1+z

S5.5. Triamterene and pimelic acid (1d·DMSO)

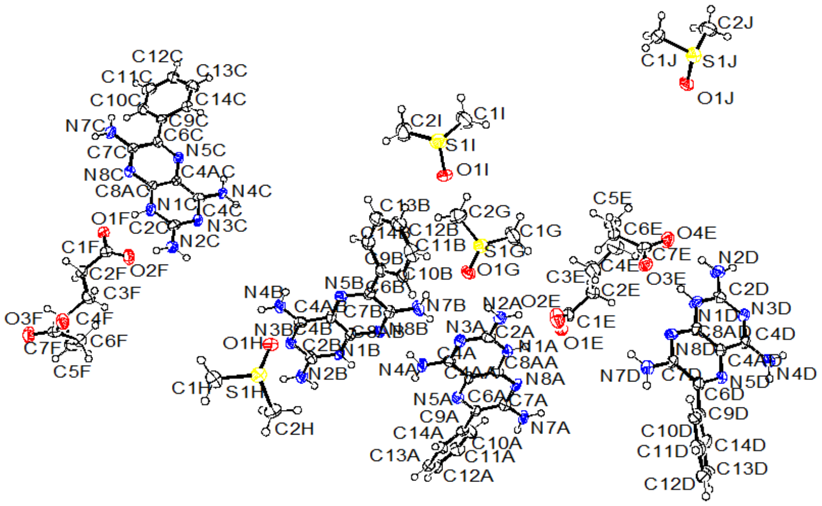

Figure S6 *ORTEP* diagram drawn at 50% probability for **1d**·DMSO.

Table S9 Hydrogen bond table for **1d**·DMSO.

No.	Type	Res	Donor--H…A	[ARU]	D--H	H…A	D…A	D--H…A
1		1	N1A—H1XA…O1E	[1555.05]	0.88	1.87	2.729(7)	166
2		2	N1B—H1XB…O4F	[1565.06]	0.88	1.84	2.707(6)	170
3		1	N2A—H2A…O2E	[1555.05]	0.88	1.80	2.654(7)	162
4		2	N2B—H2B…O3F	[1565.06]	0.88	1.91	2.743(6)	158
5		3	N2C—H2C…O2F	[1555.06]	0.88	1.79	2.657(7)	167
6		4	N2D—H2D…O4E)	[1555.05]	0.88	1.83	2.674(6)	160
7		3	N1C—H1XC…O1F	[1555.06]]	0.88	1.90	2.770(7)	168
8		1	N2A—H3A…O1G	[1555.07]	0.88	2.12	2.964(6)	161
9		2	N2B—H3B…O1J	[2566.10]	0.88	2.07	2.907(7)	159
10		3	N2C—H3C…O1H	[1566.08]	0.88	2.11	2.966(6)	163
11		4	N2D—H3D…S1I	[1565.09]	0.88	2.72	3.360(6)	131
12		4	N2D—H3D…O1I	[1565.09]	0.88	2.05	2.881(7)	156’
13		4	N1D—H1XD…O3E	[1555.05]	0.88	1.91	2.757(7)	162
14		1	N4A—H4A…N8B	[1555.02]	0.88	2.17	3.047(6)	176
15		2	N4B—H4B…N8A	[1545.01]	0.88	2.21	3.075(7)	170
16		3	N4C—H4C…N8D	[1546.04]	0.88	2.20	3.069(6)	170
17		4	N4D—H4D…N8C	[1574.03]	0.88	2.24	3.118(7)	176
18		1	N4A—H5A…O4F	[1565.06]	0.88	2.10	2.704(7)	125
19	INTRA	1	N4A—H5A…N5A	[ ]	0.88	2.45	2.778(6)	103’
20		2	N4B—H5B…O1E	[1545.05]	0.88	2.13	2.739(7)	126
21	INTRA	2	N4B…H5B…N5B	[ ]	0.88	2.52	2.827(7)	101’
22		3	N4C—H5C…O3E	[1546.05]	0.88	2.11	2.712(7)	125
23	INTRA	3	N4C—H5C…N5C	[ ]	0.88	2.49	2.804(6)	102’
24		4	N4D—H5D…O1F	[1574.06]	0.88	2.15	2.748(7)	125
25	INTRA	4	N4D—H5D…N5D	[ ]	0.88	2.50	2.817(7)	102’
26		1	N7A—H6A…O1J	[2576.10]	0.88	2.38	3.036(7)	132
27		2	N7B—H6B…O1G	[1555.07]	0.88	2.19	2.889(7)	136
28		3	N7C—H6C…O1J	[1546.09]	0.88	2.31	2.990(7)	134
29		4	N7D—H6D…O1H	[1565.08]	0.88	2.09	2.807(7)	138
30		1	N7A—H7A…N3B	[1565.02]	0.88	2.17	3.048(7)	173
31		2	N7B—H7B…N3A	[1555.01]	0.88	2.10	2.977(6)	175
32		3	N7C—H7C…N3D	[1536.04]	0.88	2.16	3.038(7)	174
33		4	N7D—H7D…N3C	[1564.03]	0.88	2.09	2.965(6)	172
34		8	C2H—H1H…O1H	[2455.08]	0.98	2.45	3.414(9)	167
35		9	C1I—H1I…O2F	[2556.06]	0.98	2.47	3.185(9)	130
36		10	C1J—H3J…O3F	[2556.06]	0.98	2.46	3.185(8)	130
37		7	C2G—H4G…O1G	[2566.07]	0.98	2.48	3.453(9)	171
38		10	C2J—H5J…N5C	[1664.03]	0.98	2.61	3.493(9)	150
39		9	C2I—HH6I…O4E	[1454.05]	0.98	2.26	3.227(9)	168
40		10	C2J—H6J…O2E	[1655.05]	0.98	2.56	3.037(9)	113
41		4	C10D—H10D…O4E	[2575.05]	0.95	2.53	3.451(8)	164
42		2	C12B—H12B…O1I	[1555.09]	0.95	2.53	3.417(9)	155
43		3	C12C—H12C…O4F	[2547.06]	0.95	2.58	3.489(9)	161

Translation of ARU-code to CIF and Equivalent Position Code:

[1565.] = [1_565] = x, 1+y, z

[2576.] = [2_576] = -x, 2-y, 1-z

[2566.] = [2_566] = -x, 1-y, 1-z

[1545.] = [1_545] = x, -1+y, z

[1556.] = [1_556] = -x, y, 1+z

[1546.] = [1_546] = x, -1+y, 1+z

[1536.] = [1_536] = x, -2+y, 1+z

[2547.] = [2_547] = -x, -1-y, 2-z

[1574.] = [1_574] = x, 2+y, -1+z

[1564.] = [1_564] = x, 1+y, -1+z

[2575.] = [2_575] = -x, 2-y, -z

[2455.] = [2_455] = -1-x, -y, -z

[2556.] = [2_556] = -x, -y, 1-z

[1664.] = [1_664] = 1+x, 1+y, -1+z

[1655.] = [1_655] = 1+x, y, z

S5.6. Triamterene and azelaic acid (1e·DMSO)

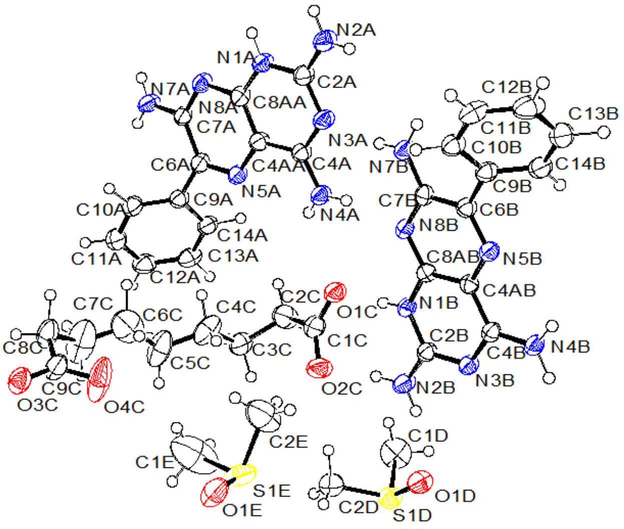

Figure S7 *ORTEP* diagram drawn at 50% probability for **1e**·DMSO.

Table S10 Hydrogen bond table for **1e**·DMSO.

No.	Type	Res	Donor--H…A	[ARU]	D--H	H…A	D…A	D--H…A
1		1	N1A—H1XA…O3C	[4464.03]	0.90(4)	1.82(4)	2.713(3)	178(4)
2		2	N1B—H1XB…O1C	[1555.03]	0.82(3)	1.89(3)	2.710(3)	174(4)
3		1	N2A—H2A…O4C	[4464.03]	0.87(4)	1.78(4)	2.650(5)	175(3)
4		2	N2B—H2B…O2C	[1555.03]	0.90(4)	1.80(4)	2.696(4)	175(4)
5		1	N2A…H3A…O1E	[4464.05]	0.79(4)	2.17(4)	2.906(4)	156(4)
6		2	N2B—H3B…O1D	[1555.04]	0.85(3)	2.06(3)	2.879(4)	161(3)
7		1	N4A—H4A…N8B	[1555.02]	0.85(3)	2.29(3)	3.128(4)	166(2)
8		2	N4B—H4B…N8A	[1655.01]	0.97(4)	2.19(4)	3.145(4)	170(3)
9		1	N4A—H5A…O1C	[1555.03]	0.81(3)	2.41(3)	2.893(3)	119(3)
10	INTRA	1	N4A—H5A…N5A	[ ]	0.81(3)	2.44(3)	2.784(4)	107(3)’
11		2	N4B—H5B…O3C	[4564.03]	0.87(4)	2.18(4)	2.788(3)	126(4)
12		1	N7A—H6A…O1D	[1455.04]	0.90(3)	2.24(3)	2.953(4)	136(3)
13		2	N7B—H6B…O1E	[4464.05]	0.86(4)	2.12(3)	2.869(4)	146(3)
14		1	N7A—H7A…N3B	[1455.02]	0.79(4)	2.24(4)	3.020(4)	175(3)
15		2	N7B—H7B…N3A	[1555.01]	0.88(3)	2.10(3)	2.973(4)	172(3)
16	INTRA	3	C3C—H4C…O2C	[ ]	1.02(3)	2.53(3)	2.895(5)	100.4(17)
17		4	C2D—H4D…O2C	[1555.03]	1.04(3)	2.36(3)	3.360(5)	163(3)
18		5	C2E—H6E…O2C	[1555.03]	1.11(6)	2.38(5)	3.461(7)	167(4)
19	INTRA	3	C6C—H10C…O4C	[ ]	1.07(4)	2.24(4)	3.031(6)	129(3)
20		1	C14A—H14A…O4C	[2545.03]	1.01(3)	2.52(3)	3.470(5)	157(2)

Translation of ARU-code to CIF and Equivalent Position Code:

[4464.] = [4_464] = -1/2+x, 3/2-y, -1/2+z

[1455.] = [1_455] = -1+x, y, z

[1655.] = [1_655} = 1+x, y, z

[4564.] = [4_564] = ½+x, 3/2-y, -1/2+z

S5.7. Triamterene and nicotinic acid (1f·DMSO)

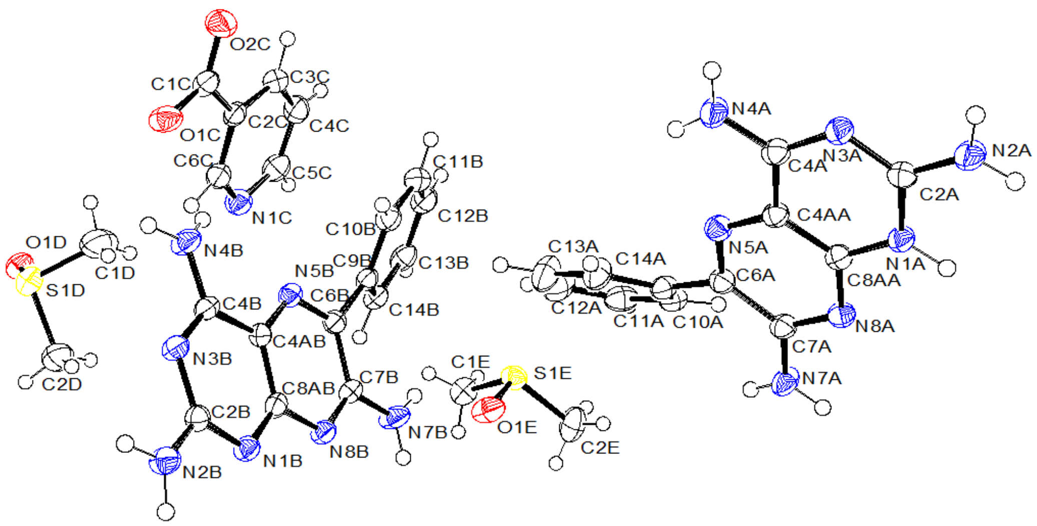

Figure S8 *ORTEP* diagram drawn at 50% probability for **1f**·DMSO

Table S11 Hydrogen bond table for **1f**·DMSO

No.	Type	Res	Donor--H...A	[ARU]	D--H	H...A	D...A	D--H...A
1		1	N1A—H1XA…O1C	[4564.03]	0.96(2)	1.72(2)	2.6638(19)	171.8(19)
2		1	N2A—H2A…O2C	[4564.03]	0.91(2)	1.88(2)	2.788(2)	178(2)
3		2	N2B—H2B…N1C	[3666.03]	0.92(2)	2.17(2)	3.079(2)	169.8(19)
4		1	N2A—H3A…O1E	[4464.05]	0.834(18)	2.039(18)	2.863(2)	169.7(16)
5		2	N2B—H3B…O1D	[1565.04]	0.90(2)	2.193(19)	3.071(2)	164.1(17)
6		1	N4A—H4A…N8B	[4464.02]	0.91(2)	2.05(2)	2.954(2)	171.4(17)
7		2	N4B—H4B…N8A	[4465.01]	0.92(2)	2.20(2)	3.106(2)	168.8(17)
8	INTRA	1	N4A—H5A…N5A	[ ]	0.83(2)	2.447(19)	2.775(2)	104.5(15)
9		2	N4B—HH5B…O1C	[1555.03]	0.880(18)	2.234(18)	2.831(2)	124.9(14)
10	INTRA	2	N4B—H5B…N5B	[ ]	0.880(18)	2.453(17)	2.790(2)	103.3(13)’
11		1	N7A—H6A…O1D	[4554.04]	0.890(19)	2.187(18)	2.885(2)	134.9(15)
12		2	N7B—H6B…O1E	[1555.05]	0.796(18)	2.202(18)	2.836(2)	136.9(16)
13		1	N7A—H7A…N3B	[4564.02]	0.88(2)	2.30(2)	3.170(2)	171.7(19)
14		2	N7B—H7B…N3A	[4565.01]	0.85(2)	2.13(2)	2.984(2)	174.2(19)
15		5	C1E—H1E…O1D	[3656.04]	0.92(2)	2.59(2)	3.412(3)	149.0(19)
16	INTRA	3	C3C—H3C…O2C	[ ]	0.952(17)	2.490(17)	2.815(2)	100.0(12)
17		2	C14B--H14B…O1D	[3656.04]	0.984(17)	2.536(2)	3.353(2)	140.4(13)
18		4	C2D—H4D…O2C	[4555.03]	0.95(3)	2.60(3)	3.421(3)	145(2)

Translation of ARU-code to CIF and Equivalent Position Code:

[4564.] = [4_564] = -1/2+x, 3/2-y, -½+z

[4464.] = [4_464] = -1/2+x, 3/2-y, -1/2+z

[4554.] = [4_554] = ½+x, ½-y, -1/2+z

[1565.] = [1_565] = x, 1+y, z

[3666.] = [3_666] = 1-x, 1-y, 1-z

[4465.] = [4_465] = -1/2+x, 3/2-y, ½+z

[4565.] = [4_565] = ½+x, 3/2-y, ½+z

[3656.] = [3_656] = 1-x, -y, 1-z

[4555.] = [4_555] = 1/2+x, 1/2-y, 1/2+z

S5.8. Triamterene and Ibuprofen (1g·DMSO)

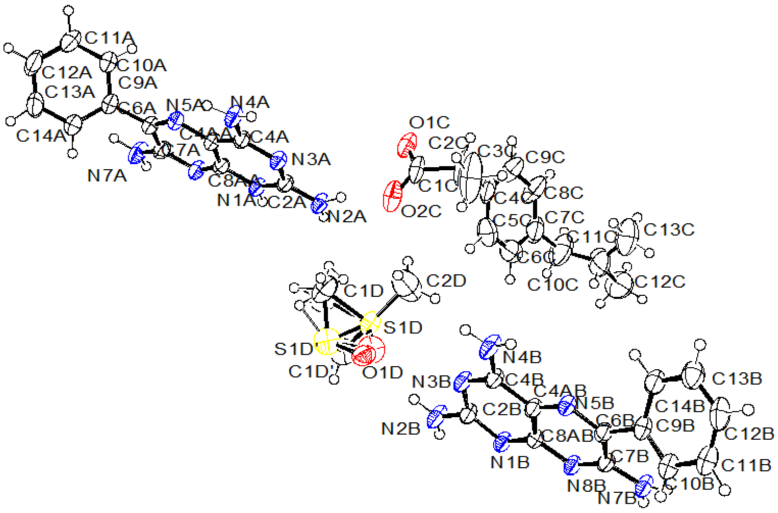

Figure S9 *ORTEP* diagram drawn at 50% probability for **1g**·DMSO.

Table S12 Hydrogen bond table for **1g**·DMSO.

No.	Type	Res	Donor--H…A	[ARU]	D--H	H…A	D…A	D--H…A
1		1	N2A—H2A…N1B	[2666.02]	0.85(3)	2.02(3)	2.857(3)	166(3)
		2	N2B—H2B…?	[ ]	0.80(4)			
2		1	N2A—H3A…O2C	[1555.03]	0.90(4)	1.89(4)	2.774(4)	165(3)
3		2	N2B—H3B…O1D	[1555.04]	0.88(3)	2.15(3)	3.004(6)	163(3)
4		1	N4A—H4A…N3A	[2665.01]	0.88(4)	2.14(4)	3.006(3)	169(3)
5		2	N4B—H4B…N3B	[2766.02]	0.87(4)	2.14(4)	3.006(3)	173(3)
6	INTRA	1	N4A—H5A…N5A	[ ]	0.91(4)	2.50(4)	2.797(4)	100(2)
7		1	N4A—H5A…O2C	[2665.03]	0.91(4)	2.27(4)	2.956(4)	132(3)’
8	INTRA	2	N4B—H5B…N5B	[ ]	0.88(4)	2.49(3)	2.792(4)	101(2)
9		2	N4B—H5B…O1D	[2766.04]	0.88(4)	2.22(3)	2.927(9)	137(3)’
		1	N7A—H6A…?	[ ]	0.95(4)			
10		2	N7B—H6B…O1C	[1656.03]	0.83(3)	2.15(3)	2.817(4)	136(3)
11		1	N1A—H1XA…N8B	[2666.02]	0.86(3)	2.10(3)	2.960(3)	175(3)
12		1	N7A—H7A…O1C	[2565.03]	0.92(4)	1.88(4)	2.793(4)	171(4)
13		2	N7B—H7B…N8A	[2666.01]	0.87(3)	2.14(3)	3.001(3)	170(4)
14		3	C6C—H6C…O1D	[2666.04]	0.95	2.47	3.238(7)	138
15		4>	C2D—H6D…O2C	[1555.03]	0.98	2.33	3.266(7)	159

Translation of ARU-code to CIF and Equivalent Position Code:

[2666.] = [2_666] = 1-x, 1-y, 1-z

[2665.] = [2_665] = 1-x, 1-y, -z

[2565.] = [2_565] = --x, 1-y, -z

[2766.] = [2_766] = 2-x, 1-y, 1-z

[1656.] = [1_656] = 1+x, y, 1+z

S6. Experimental (LAG) and simulated PXRD patterns for 1a – g·DMSO

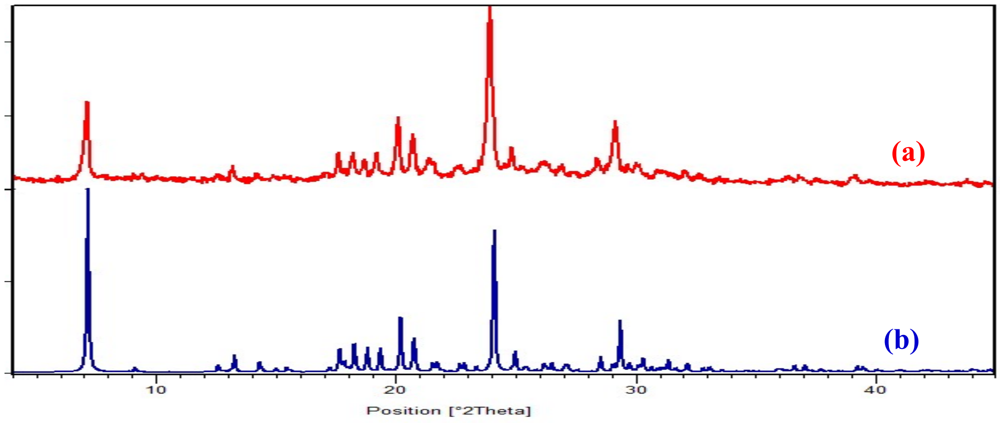

Figure S10 Comparison of the PXRD pattern for adduct **1a**·DMSO obtained from (a) LAG with (b) simulated from X-ray structure.

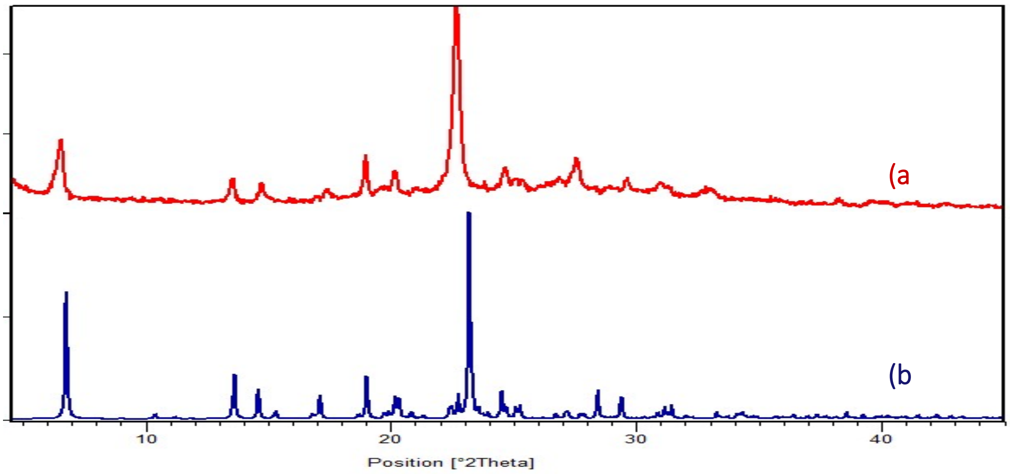

Figure S11 Comparison of the PXRD pattern for the adduct **1b**·DMSO obtained from (a) LAG with (b) simulated from X-ray structure.

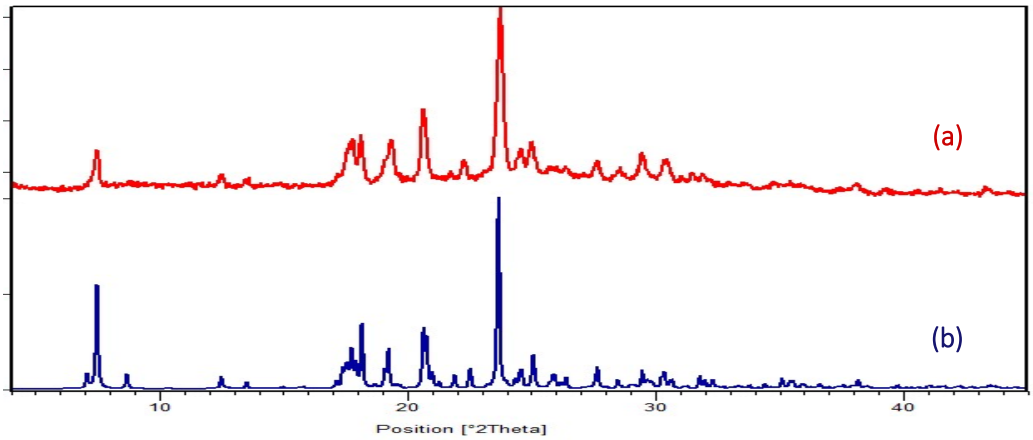

Figure S12 Comparison of the PXRD pattern for the adduct **1c**·DMSO obtained from (a) LAG with (b) simulated from X-ray structure.

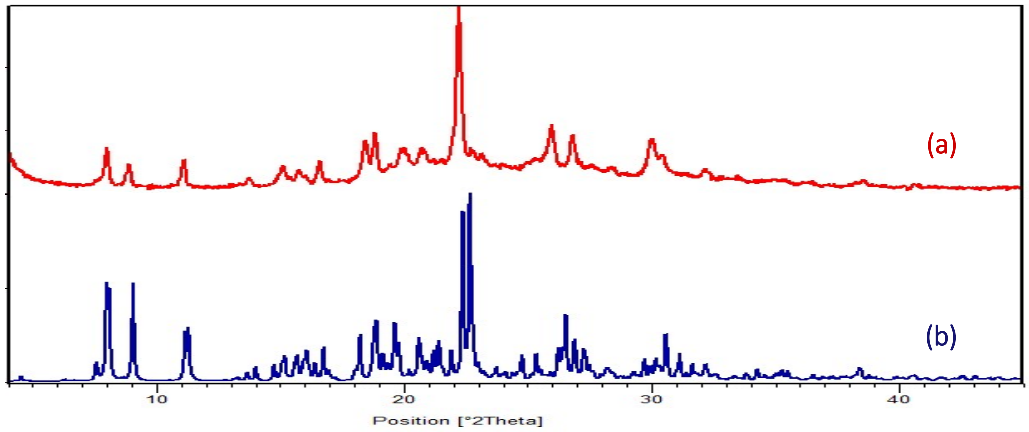

Figure S13 Comparison of the PXRD pattern for the adduct **1d**·DMSO obtained from (a) LAG with (b) simulated from X-ray structure.

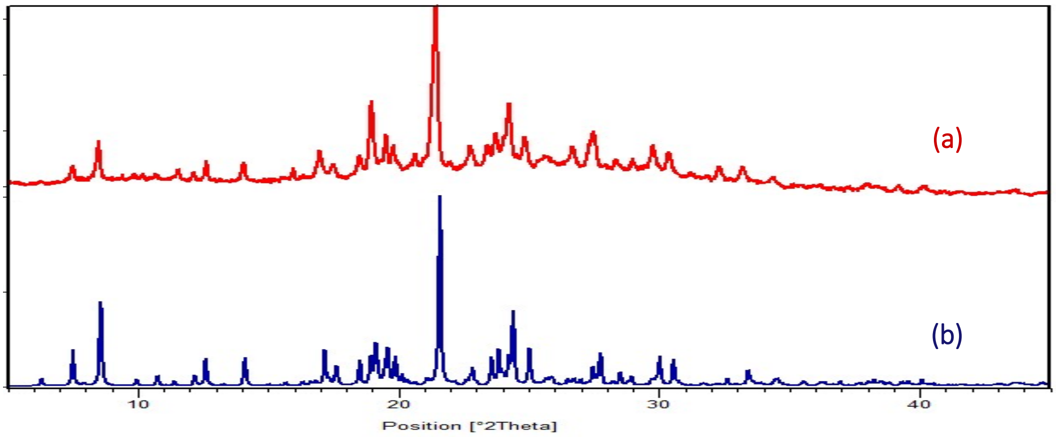

Figure S14 Comparison of the PXRD pattern for the adduct **1e**·DMSO obtained from (a) LAG with (b) simulated from X-ray structure.

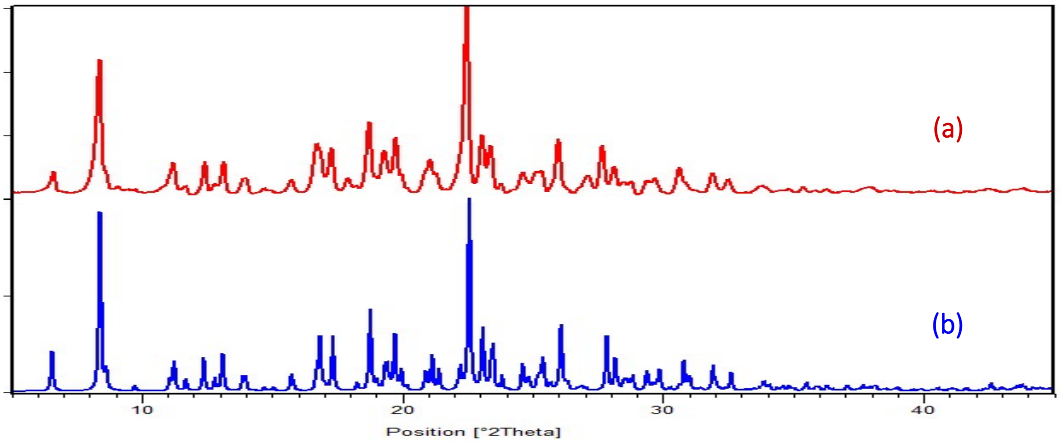

Figure S15 Comparison of the PXRD pattern for the adduct **1f**·DMSO obtained from (a) LAG with (b) simulated from X-ray structure.

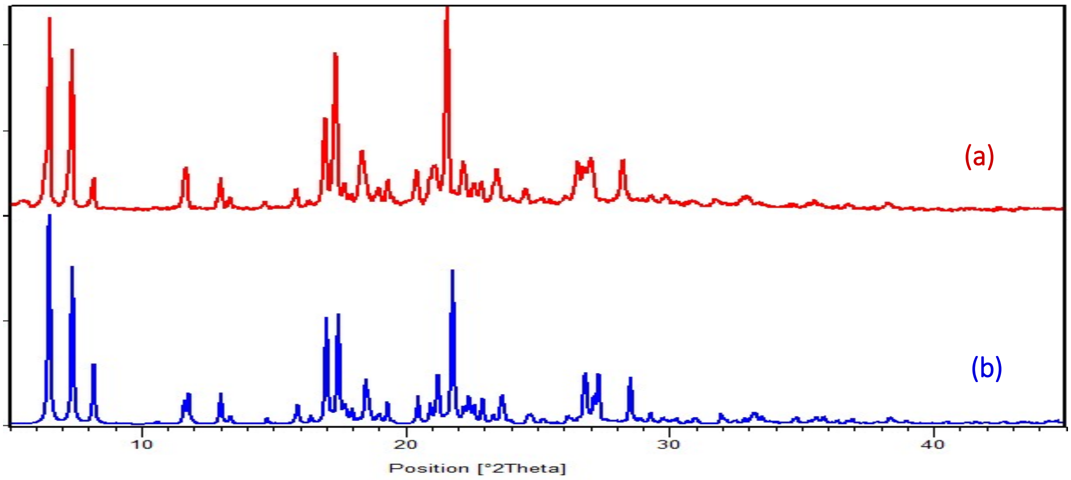

Figure S16 Comparison of the PXRD pattern for the adduct **1g**·DMSO obtained from (a) LAG with (b) simulated from X-ray structure.

Summary

These results show that the crystals grown for single crystal analysis were representative of the bulk samples **1a**–**g**·DMSO. The differences between the experimental and simulated PXRD pattern for **1d**.DMSO are thought to be due to the preferred orientation of the plate-like crystals noted in this sample.

S7. DSC and TGA of the crystalline product for 1 and 1a–g·DMSO

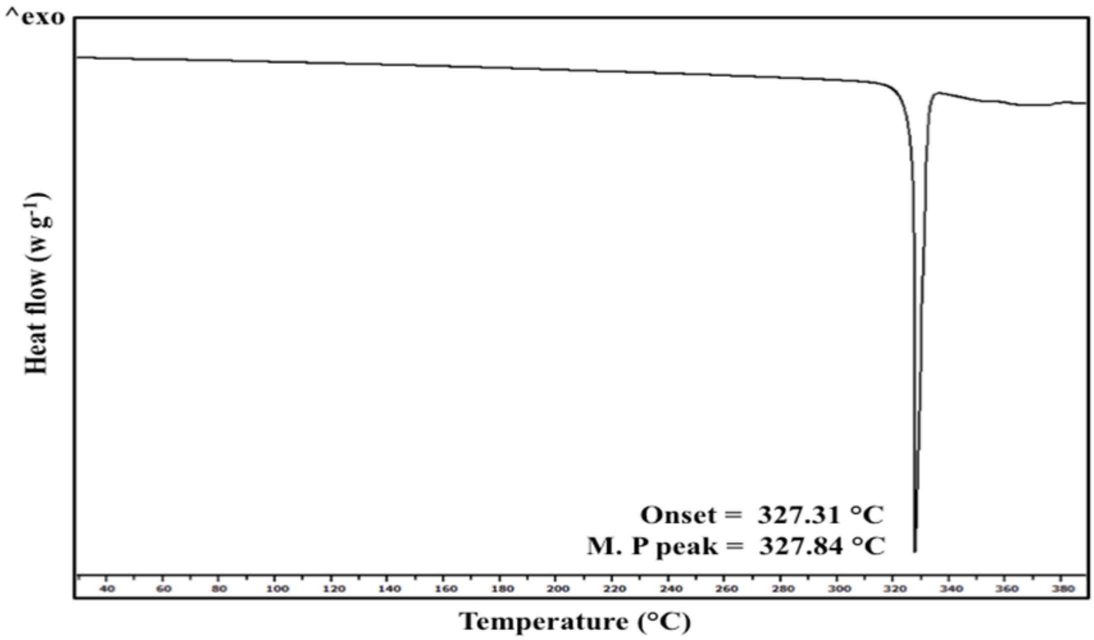

Figure S17 The DSC trace for triamterene (**1**) showing the sharp melting peak at 327.84°C (for reference).

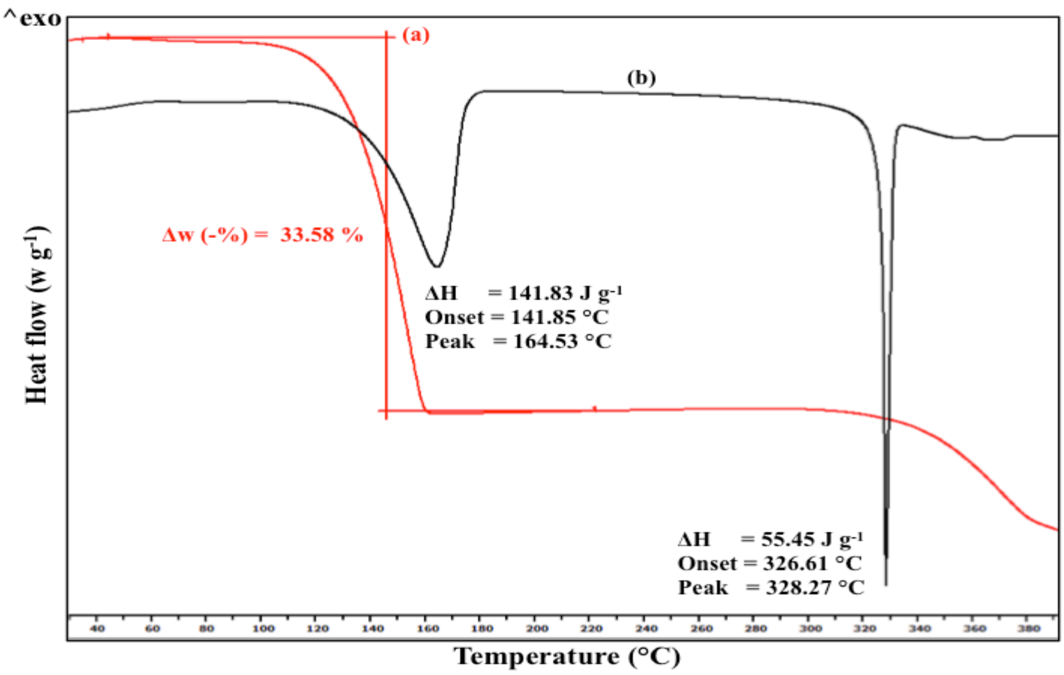

Figure S18 TGA (a) and DSC (b) trace for **1a**·DMSO showing an initial weight loss of 33.58% in the TGA corresponding to the concomitant weight loss of **a** (acetic acid) and DMSO as calculated (35.07%) from a stoichiometry of 1 : 1 : 1 (**1**: **a** : DMSO). The melting point peak of 328.27°C in the DSC corresponds to pure **1**.

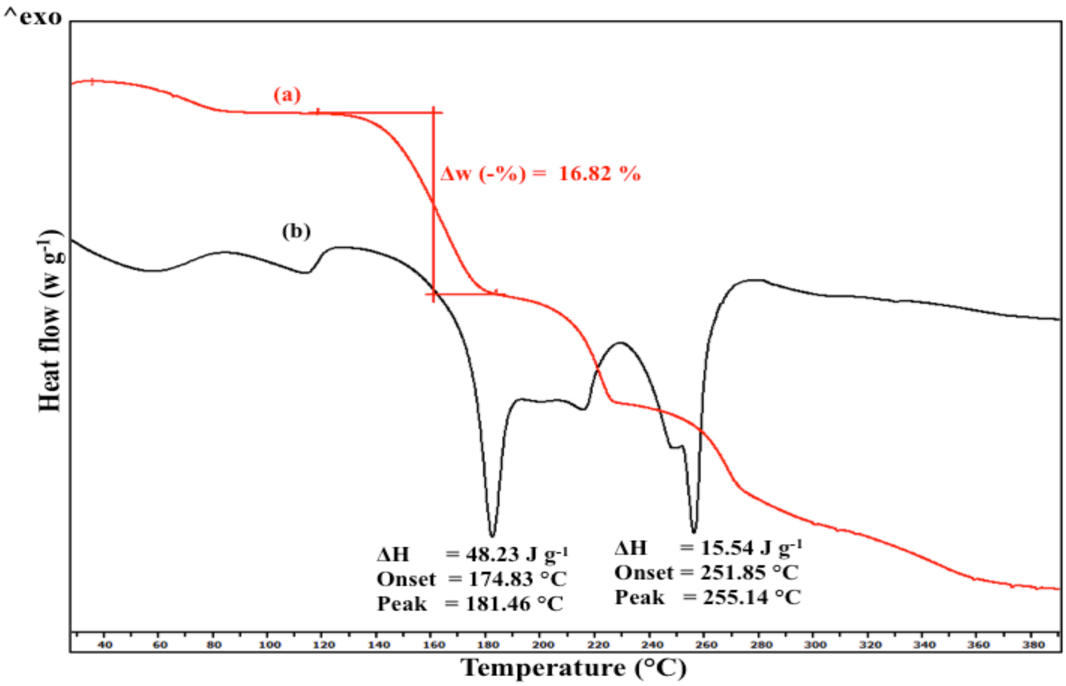

Figure S19 TGA (a) and DSC (b) trace for the adduct **1b**·DMSO. The TGA trace shows an initial weight loss which is likely due to physically absorbed solvent while second weight loss of 16.82%, related to weight of DMSO as calculated (17.38%) from a stoichiometry of 1 : 1 : 1 (**1** : **b** : DMSO). This is associated with the third endothermic event in the DSC with corresponding enthalpy change of 48.23 Jg^{-1} with an onset temperature of 174.83°C. After removal of solvent the product melts at 255.14°C.

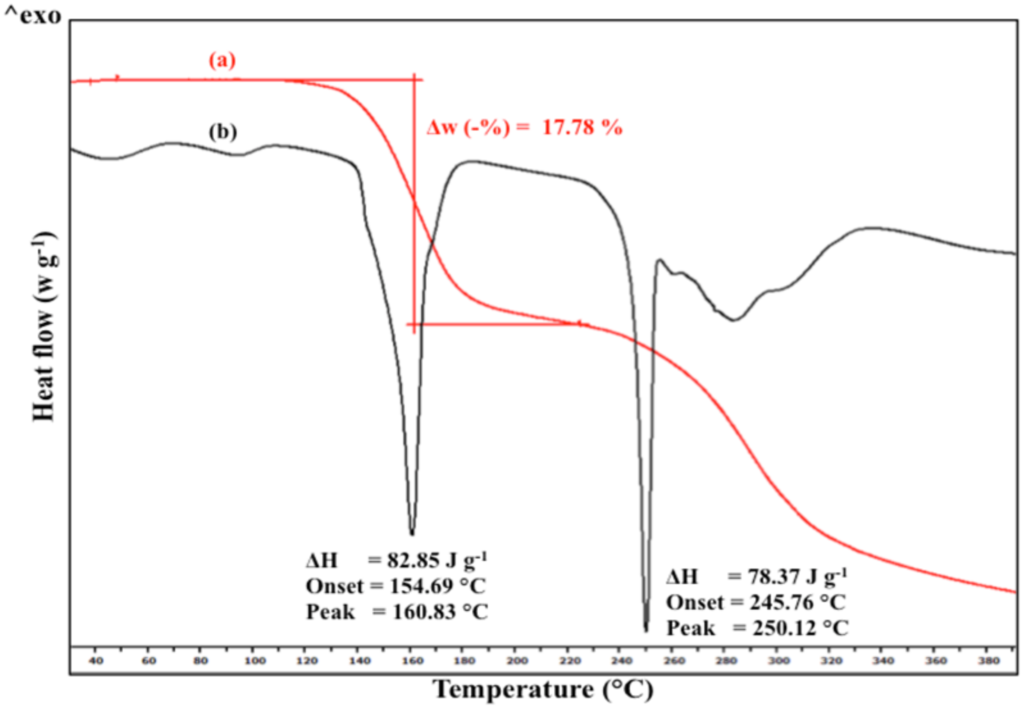

Figure S20 TGA (a) and DSC (b) trace for the adduct **1c**·DMSO. The TGA trace shows an initial weight loss of 17.78%, corresponding to calculated weight loss of DMSO (19.31%) from **1** : **c** : DMSO (2 :1 : 2 stoichiometry). The DSC trace shows the melting point peak of the product (with solvent removed) at 250.12°C.

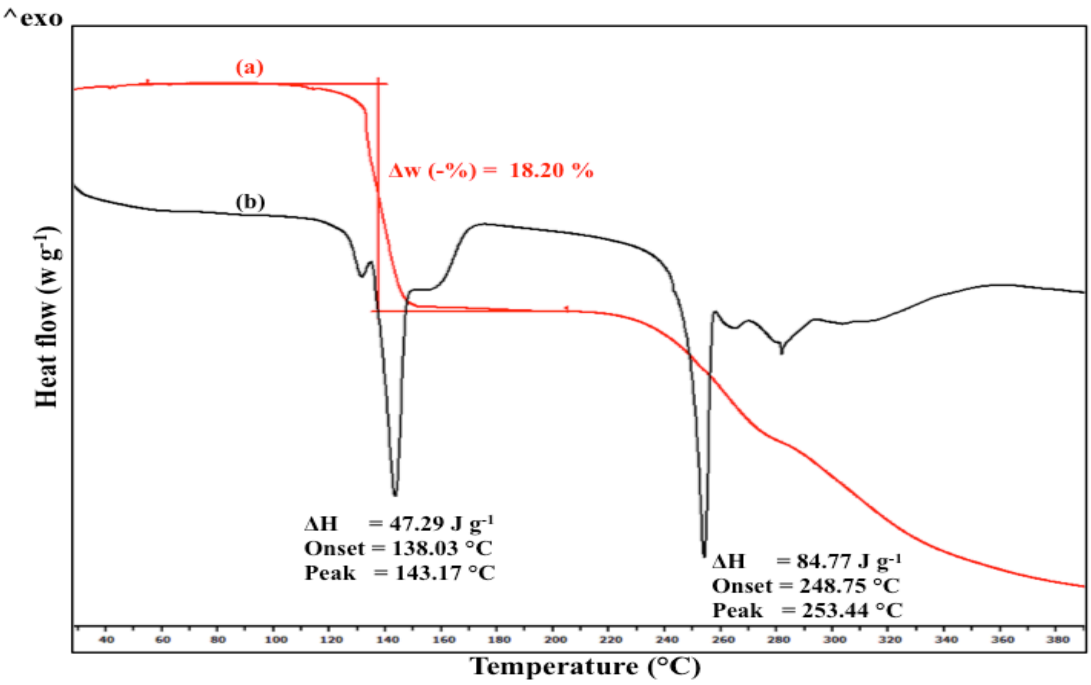

Figure S21 TGA (a) and DSC (b) trace for the adduct **1d**·DMSO. The TGA trace shows an initial weight loss of 18.20 %, corresponding to calculated weight loss of DMSO (18.98 %) from **1** : **d** : DMSO (2 : 1 : 2 stoichiometry). The DSC trace shows the melting point peak of the desolvated product at 253.44°C.

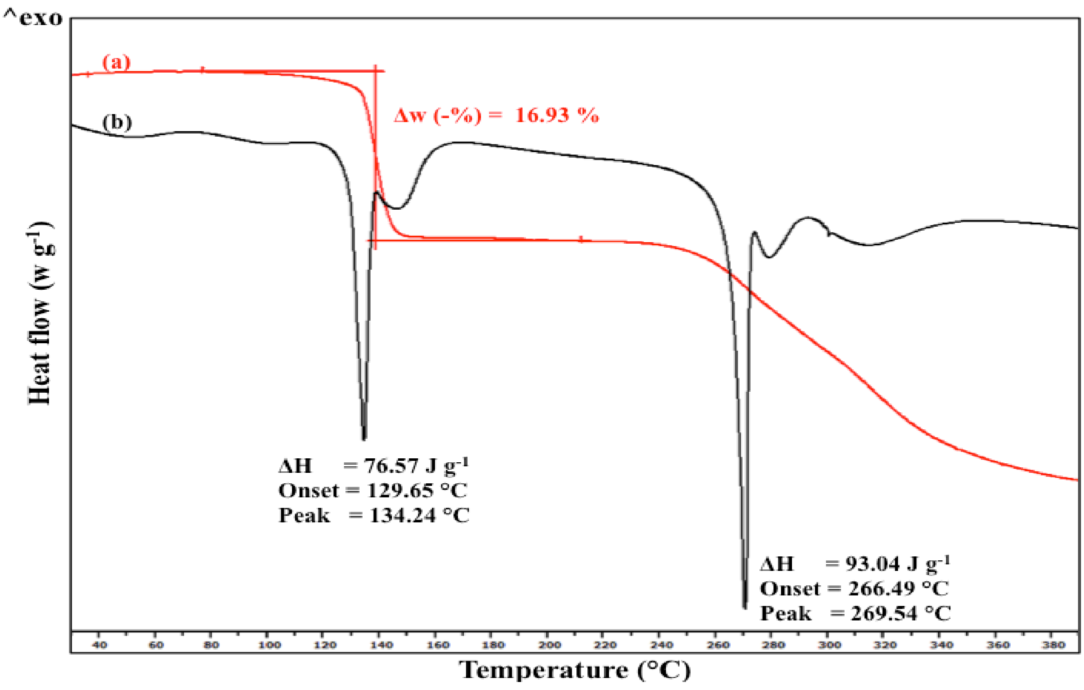

Figure S22 TGA (a) and DSC (b) trace for the adduct **1e**·DMSO. The TGA trace shows an initial weight loss of 16.93 %, corresponding to calculated weight loss of DMSO (18.36 %) from **1** : **e** : DMSO (2 : 1 : 2) stoichiometry. The DSC trace shows the melting point peak of the desolvated product at 269.54°C.

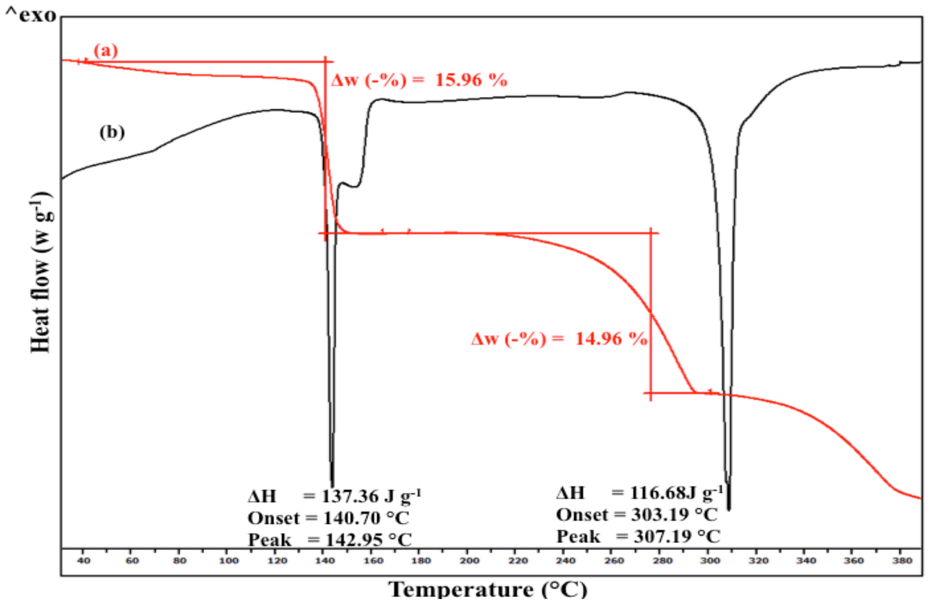

Figure S23 TGA (a) and DSC (b) trace for the adduct **1f**·DMSO. The experimental weight loss for the DMSO is found to be 16%, which is slightly less than expected (19.88%) calculated from a stoichiometry of **1** : **f** : DMSO (2 : 1 : 2), possibly due to some solvent loss to the atmosphere prior to performing the thermal analysis. This weight loss is associated with an endothermic event in the DSC with an onset temperature of 137.4 °C. After the removal of DMSO, the TGA trace shows a further weight loss of 15 %, in good agreement with the weight of nicotinic acid (15.7 %) present in the adduct **1f**·DMSO (2 : 1 : 2). After the removal of DMSO and nicotinic acid only triamterene is left, which has a melting point peak of 307.19°C.

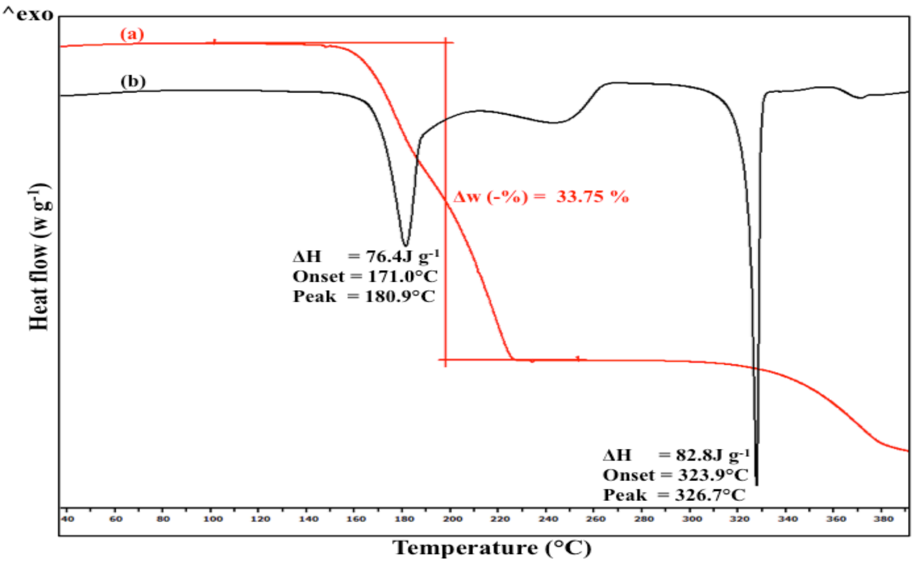

Figure S24 TGA (a) and DSC (b) trace for the adduct **1g**·DMSO. The experimental weight loss of 33.75 % in the TGA trace corresponds to the concomitant weight loss of DMSO and ibuprofen (35.95 %) calculated from a stoichiometry of 2 : 1 : 1 for **1g**·DMSO. The first endotherm in the DSC scan with an onset temperature of 171.0°C is related with DMSO loss and second broad endothermic event indicates the degradation and/or removal of ibuprofen. After the removal of DMSO and ibuprofen triamterene is left which gives a melting point peak at 326.7°C.

Table S13 Summary of data obtained from DSC/TGA curves

Triamterene or Adduct	Melting (or boiling where relevant) point of coformer from the literature (°C)	Melting point onset of first endotherm (°C)	Calculated weight loss (%)	Experimental weight loss (%)	Melting point onset of final product (°C)
**1**	N/A	N/A	N/A	N/A	327.31
**1a**·DMSO	16.2<br>(117–118)	141.85	35.07	33.58	326.61
**1b**·DMSO	184–186	174.83	17.38	16.82	251.85
**1c**·DMSO	151–154	154.69	19.32	17.78	245.76
**1d**·DMSO	103–105	138.03	18.99	18.20	248.75
**1e**·DMSO	109–111	129.65	18.36	16.93	266.49
**1f**·DMSO	236–239	140.70	19.88(1)<br>15.54(2)	15.96(1)<br>14.96(2)	303.19
**1g**·DMSO	77–78	171.00	35.80	33.75	323.90

Table S14 Calculation of stoichiometry from TGA data

**Triamterene or Adduct**	**Calculation of stoichiometry using TGA data (Triamterene : Coformer : Solvent)**
**1**	1 : 0 : 0
**1a**·DMSO	1 : 1 : 1
**1b**·DMSO	1 : 1 : 1
**1c**·DMSO	2 : 1 : 2
**1d**·DMSO	2 : 1 : 2
**1e**·DMSO	2 : 1 : 2
**1f**·DMSO	2 : 1 : 2
**1g**·DMSO	2 : 1 : 1

S8. Summary of stoichiometry derived from SCXRD and thermal data

Table S15 Conformation of stoichiometry from SCXRD and thermal methods

Crystallization experiment	Product designation	Stoichiometry by SCXRD (Triamterene : Coformer : Solvent)	Confirmation of stoichiometry using Thermal data (Triamterene : Coformer : Solvent)
**Triamterene**	**1**	1 : 0 : 0	1 : 0 : 0
**Triamterene, acetic acid and DMSO**	**1a**·DMSO	1 : 1 : 1	1 : 1 : 1
**Triamterene, succinic acid and DMSO**	**1b**·DMSO	1 : 1 : 1	1 : 1 : 1
**Triamterene, adipic acid and DMSO**	**1c**·DMSO	2 : 1 : 2	2 : 1 : 2
**Triamterene, pimelic acid and DMSO**	**1d**·DMSO	2 : 1 : 2	2 : 1 : 2
**Triamterene, azelaic acid and DMSO**	**1e**·DMSO	2 : 1 : 2	2 : 1 : 2
**Triamterene, nicotinic acid and DMSO**	**1f**·DMSO	2 : 1 : 2	2 : 1 : 2
**Triamterene, ibuprofen and DMSO**	**1g**·DMSO	2 : 1 : 1	2 : 1 : 1

S9. References

Cruz-Cabeza, A. J. (2012). *CrystEngComm*, **14**, 6362-6365.

Farrugia, L. J. (2012). *J Appl Cryst.* **45**, 849-854.

Hughes, D. S., Delori, A., Rehman, A. & Jones, W. (2017). *Chem. Cent. J.* **11**, 63-72.

Spek, A. L. (2009). *Acta Cryst.* **D65**, 148-155.
